# Genomic characterization and evolutionary dynamics of human adenovirus C (HAdV-C) in Beijing (2023–2024): insights into multiple recombination and adaptive evolution

**DOI:** 10.1093/ve/veag040

**Published:** 2026-07-01

**Authors:** Changcheng Wu, Chen Mai, Zhihao Zhao, Shiyao Zhang, Lin Ma, Fei Ye, Weibang Huo, Yuda Chen, Meihui Luo, Xiaona Yang, Wenling Wang, Jiegang Huang, Wenjie Tan

**Affiliations:** National Key Laboratory of Intelligent Tracking and Forecasting for Infectious Diseases, National Institute for Viral Disease Control and Prevention, Chinese Center for Disease Control and Prevention, Beijing 102206, China; National Key Laboratory of Intelligent Tracking and Forecasting for Infectious Diseases, National Institute for Viral Disease Control and Prevention, Chinese Center for Disease Control and Prevention, Beijing 102206, China; School of Public Health, Guangxi Medical University, Nanning 530021, Guangxi, China; Guangxi Key Laboratory of AIDS Prevention and Treatment, Guangxi Medical University, Nanning 530021, Guangxi, China; National Key Laboratory of Intelligent Tracking and Forecasting for Infectious Diseases, National Institute for Viral Disease Control and Prevention, Chinese Center for Disease Control and Prevention, Beijing 102206, China; Beijing Chaoyang District Center for Disease Control and Prevention, No. 25 Hujing Road, Chaoyang District, Beijing 100021, China; Beijing Daxing District Center for Disease Control and Prevention, No. 55 Xinghua Street, Daxing District, Beijing 102600, China; National Key Laboratory of Intelligent Tracking and Forecasting for Infectious Diseases, National Institute for Viral Disease Control and Prevention, Chinese Center for Disease Control and Prevention, Beijing 102206, China; National Key Laboratory of Intelligent Tracking and Forecasting for Infectious Diseases, National Institute for Viral Disease Control and Prevention, Chinese Center for Disease Control and Prevention, Beijing 102206, China; Beijing Chaoyang District Center for Disease Control and Prevention, No. 25 Hujing Road, Chaoyang District, Beijing 100021, China; Beijing Daxing District Center for Disease Control and Prevention, No. 55 Xinghua Street, Daxing District, Beijing 102600, China; Beijing Daxing District Center for Disease Control and Prevention, No. 55 Xinghua Street, Daxing District, Beijing 102600, China; National Key Laboratory of Intelligent Tracking and Forecasting for Infectious Diseases, National Institute for Viral Disease Control and Prevention, Chinese Center for Disease Control and Prevention, Beijing 102206, China; School of Public Health, Guangxi Medical University, Nanning 530021, Guangxi, China; Guangxi Key Laboratory of AIDS Prevention and Treatment, Guangxi Medical University, Nanning 530021, Guangxi, China; National Key Laboratory of Intelligent Tracking and Forecasting for Infectious Diseases, National Institute for Viral Disease Control and Prevention, Chinese Center for Disease Control and Prevention, Beijing 102206, China

**Keywords:** HAdV-C108, recombination, genomic evolution, phylodynamic, respiratory infection, positive selection

## Abstract

Human adenovirus type C (HAdV-C) causes upper respiratory infections in children and may lead to severe pneumonia. During the implementation and subsequent relaxation of non-pharmaceutical interventions, HAdV-C emerged as a transiently dominant circulating strain in Beijing. However, the fine-scale genomic architecture and the evolutionary trajectories governing its recombination remains insufficiently characterized. Between March 2023 and August 2024, respiratory samples from Beijing patients were collected and screened by quantitative PCR. In conjunction with high-throughput whole-genome sequencing, five complete HAdV-C genomes were characterized, predominantly identified as genotypes C1 and C108, maintaining over 98% intra-typic sequence identity. Phylogenetic analysis based on whole genomic sequences revealed at least five evolutionary branches within C108. Phylogenetic reconstruction revealed a complex diversification within C108, delineating at least five distinct evolutionary clades. Specifically, the four C108 strains partitioned into two divergent sub-lineages, exhibiting close phylogenetic affinities with sequences from China and the United States. Furthermore, recombination analysis identified six discrete recombination patterns. Selection pressure analysis further demonstrated heterogenous evolutionary constraints across the genome; notably, immune-relevant early genes such as *E1a_26KD* exhibited elevated *dN/dS* ratios, harbouring multiple positive selection sites. These adaptive mutations were distributed across 23 of 33 annotated genes (69.7%), suggesting extensive diversifying selection. These findings elucidate that HAdV-C evolution is synergistically driven by frequent recombination events and potent selective pressures. This study provides critical evidence for the spatiotemporal dynamics and genomic surveillance of the emerging HAdV-C variants.

## 1. Introduction

Human adenovirus (HAdV) belongs to the genus *Mastadenovirus* within the family *Adenoviridae*. It is a non-enveloped, double-stranded DNA virus with a genome length of ~34–36 kb (Robinson et al. 2013). HAdV encodes ~30 proteins involved in genome replication, metabolic reprogramming, structural formation, and immune regulation (Russell 2009, Sanchez et al. 2025). The virion exhibits an icosahedral structure, and its capsid is primarily composed of three structural proteins: penton base, hexon, and fiber (Gallardo et al. 2021). HAdV demonstrates strong tissue tropism and pathogenicity, capable of causing multisystem infections involving the respiratory (Jianhua et al. 2025), gastrointestinal (Kajon 2024), urinary, and ocular tracts (Zhao et al. 2022). In immunocompromised individuals, HAdV infections could lead to severe and even fatal complications (Liang et al. 2025). To date, at least 116 HAdV genotypes have been identified (HAdV Working Group) and classified into seven groups (A–G) based on sequence homology (Zang et al. 2025, Dadkhah et al. 2026). Among these, HAdV-C—encompassing genotypes 1, 2, 5, 6 and 57—is recognized as a ubiquitous pathogen responsible for acute respiratory infections (ARIs) in children (Mao et al. 2022). Recombination is a known driver of diversity within HAdV-C (Dadkhah et al. 2026). For example, a recent study utilizing whole genome sequencing (WGS) of HAdV-C isolated from wastewater in Tianjin, China, identified significant recombination events within the *hexon* and *fiber* genes (Lei et al. 2023). Besides, Wang *et al*. recently reported C108 which was derived from both type C1 and C2 had been detected in China in past two decades (Wang et al. 2025). However, the fine-scale evolutionary dynamics of these recombinants remain to be fully elucidated. This finding challenged earlier perspectives which suggested that recombination in the major capsid genes (*penton base*, *hexon* and *fiber*) was uncommon in HAdV-C compared to other genotypes (Ismail et al. 2018).

Before 2021, genotypes B3 and B7 of HAdV were responsible for 56.57% and 32% of ARIs among children in Beijing, respectively, whereas HAdV-C1 accounted for only 6% (Wang et al. 2024). Beijing implemented its most stringent non-pharmaceutical interventions (NPIs) on 24 January 2020, encompassing mandatory stay-at-home orders, the suspension of large-scale public gatherings, and localized lockdowns in affected areas (Li et al. 2020). Following the widespread adoption of NPIs, the total HAdV positivity rate declined by 55.8% (from 3.19% to 1.41%) (Wang et al. 2023). During this period, the prevalent HAdV-B3 and B7 decreased markedly—with the latter becoming virtually undetectable—while HAdV-C1 established dominance, accounting for 39.3% of infections (Wang et al. 2023).

As the COVID-19 pandemic was brought under control, China rescinded most control measures on 7 December 2022. Recent surveillance indicates that during 2023–2024, the predominant circulating HAdV strains shifted transiently from type B to C in several regions (Wang et al. 2023, Niu et al. 2026, Jin et al. 2025, Wu et al. 2025). Consequently, analysing HAdV-C sequences from this post-NPI window is crucial for understanding the shifting evolutionary trajectories and circulation patterns of the virus. These changes underscore the necessity of enhanced surveillance of HAdV-C. Despite this necessity, comprehensive genomic studies on the selection characteristics and recombination-driven evolution of HAdV-C in Beijing remain sparse. The lack of localized genomic surveillance may compromise the accuracy of molecular diagnostics and the efficacy of early surveillance systems (Zhang et al. 2024).

In this study, we analysed five HAdV-C genome sequences obtained from patients with upper respiratory tract infections in Beijing between March 2023 and August 2024. Through phylogenetic, recombination and selection analyses, we aimed to elucidate the genomic features and evolutionary dynamics of these local strains. Our findings identify complex recombination and adaptive mutations as key factors driving HAdV-C diversity, providing critical data to support molecular surveillance and public health interventions.

## 2. Materials and methods

### 2.1. Sample collection

This study was conducted in accordance with relevant guidelines and regulations. Ethical approval was granted by National Institute for Viral Disease Control and Prevention, Chinese Center for Disease Control and Prevention (Ethics Approval No. IVDC2024–028). Patients diagnosed with ARIs were enrolled from four medical institutions in Beijing between March 2023 and August 2024. Throat swab specimens were collected by healthcare professionals following standardized protocols. All 47 samples were initially screened using a Respiratory 17 Types Pathogen Multiplex Nucleic Acid Detection Kit (TianLong, China). Specimens testing positive for HAdV were subsequently confirmed using a human adenovirus (ad) PCR Detection Kit (TianLong, China) on a SLAN-96P real-time fluorescence quantitative PCR system, with the positivity threshold defined as a cycle threshold (*C_t_*) value *<* 30.

### 2.2. Library construction and sequencing

Following DNA extraction, sequencing libraries were constructed using the MGIEasy Fast Enzymatic Digestion Library Construction Kit V2.0 (MGI Tech, China), strictly adhering to the manufacturer’s instructions. The prepared libraries were then processed into DNA nanoballs (DNBs) using the DNBSEQ One-Step DNB Preparation Kit V2.0 (MGI Tech, China). DNB concentrations were quantified using the Qubit ssDNA Assay Kit (Thermo Fisher Scientific, USA), and libraries meeting the concentration threshold of ≥4 ng/μl were selected for sequencing. High-throughput sequencing was performed on the MGISEQ-200RS (MGI Tech, China) platform using a 150 bp paired-end (PE150) strategy.

### 2.3. Genome assembly

Raw sequencing reads were quality-controlled using fastp v0.23.2 (Chen et al. 2018). To remove host contamination, reads were aligned to the human reference genome (GRCh38/hg38) using BWA v0.7.17 (Li and Durbin 2009). Non-host reads were taxonomically classified and annotated using Kraken2 v2.1.2 (Wood et al. 2019) combined with Bracken (Lu et al. 2017) for relative abundance estimation. *De novo* assembly of non-host reads was performed using SPAdes v3.15.3 (Bankevich et al. 2012). Genotypes were determined by aligning contigs against a curated HAdV reference database using blastn v2.12.0+ (Camacho et al. 2009). Consequently, AF534906.1 and JX173081.1 were utilized as references for the C1 and C2 strains. Subsequently, raw sequencing reads were mapped to the most closely related reference sequence. Variant calling was executed using FreeBayes v1.3.6 (Garrison and Marth 2012), followed by post-calling filtration with bcftools v1.16 (Danecek et al. 2021). To ensure the high-confidence of the variants, only positions with a sequencing depth > 10× and an allele frequency > 50% were retained. These validated variants were then utilized to generate the final viral consensus genomes, with low-coverage regions (< 10×) masked with ‘N’ to prevent erroneous base calling. Sequences obtained can be found in GenBase with accession numbers C_AA134039.1-C_AA134043.1.

### 2.4. Phylogenetic analysis

HAdV-C sequences were collected from GenBank, GenBase and the National Microbiology Data Center (NMDC). After excluding low-quality sequences, 374 HAdV-C genomes were retained (Table S1). Genotype strains of all HAdV-C were included: HAdV-C1 (AF534906.1), HAdV-C2 (JX173081.1), HAdV-C5 (AY339865.1), HAdV-C6 (FJ349096.1), HAdV-C57 (HQ003817.1), HAdV-C89 (MH121097.1), HAdV-C104 (MH558113.1) and HAdV-C108 (ON054624.1). Whole-genome sequences were aligned using MAFFT v7.526 (Katoh and Standley 2013). Whole-genome, *penton base*, *hexon* and *fiber* gene sequences were used to construct maximum likelihood (ML) phylogenetic trees with iqtree2 v2.4.0 (Minh et al. 2020). According to the Bayesian information criterion, the optimal models being GTR + F + R10, HKY + F, TIM2 + F + I and TPM2 + F + I, respectively. Branch support was assessed with 1 000 bootstrap replicates, with a threshold of > 70. Tree visualization was performed using ggtree package (Yu et al. 2018).

### 2.5. Recombination and selection pressure analysis

To avoid interference from potential recombination in non-prototype sequences, only HAdV-C1, C2, C5 and C6 prototype strains were chosen as the references for recombination analysis. Recombination analysis was performed using RDP4 (Martin et al. 2015). Events were considered valid only if detected by at least five of the seven available methods. Recombination signals were verified using Simplot v3.5.1 (Lole et al. 1999), with the parameters set as follows: window size 5000 bp, step size 100 bp, and Kimura 2-parameter model. For selection pressure analysis, the coding sequences of the five strains were concatenated. The ratio of non-synonymous (N) to synonymous (S) substitutions was estimated using the yn00 program in PAML v4.9 (Yang 2007). To minimize computational redundancy, sequences within the same clade exhibiting over 99.5% nucleotide identity were downsampled. Only representative strains were retained, resulting in a curated dataset of 66 sequences for site-specific selection pressure assessment (Table S2). Positive selection at individual sites was evaluated using EasyCodeML v1.4 (Gao et al. 2019) under the site-specific model, employing Likelihood Ratio Tests (LRTs) to compare the neutral models with selection models (M0 versus M3, M1a versus M2a, and M7 versus M8). Sites were considered positive selection if they showed a posterior probability > 0.99 (indicated by ^**^).

### 2.6. Structure prediction and visualization

Functional domains of the E1A and E1B-55 K protein were predicted *via* InterPro (Blum et al. 2025), and protein structures were modelled using AlphaFold3 (Abramson et al. 2024) and visualized using PyMOL (http://www.pymol.org). Positive selection sites were visualized using Snipit v1.7 (O'Toole et al. 2024).

### 2.7. Estimation of the time to the most recent common ancestor

To estimate the time to the most recent common ancestor (tMRCA), we curated a dataset comprising whole-genome sequences of HAdV-C1 and C108 collected from 2010 onwards. The temporal signal of the sequence data was initially rigorously assessed using TempEst v1.5.3 (Rambaut et al. 2016) *via* root-to-tip regression analysis. The R^2^ values of C1 and C108 were 0.19 and 0.4, respectively. Subsequently, Bayesian phylogenetic inference was performed using BEAST X v10.5.0 (Baele et al. 2025). The Bayesian SkyGrid model was employed, a non-parametric tree prior that better accommodated complex population dynamics (Mah et al. 2024). We employed a HKY nucleotide substitution model in conjunction with a Γ + *I* (Gamma + Invariant Sites) site heterogeneity model. With the 2ln (Bayes Factor) of C1 and C108 exceeded 200, an uncorrelated relaxed log-normal molecular clock was applied (Tables S3-S4). Three independent MCMC chains were executed for times 1 × 10^9^ generations each, with parameters sampled every 1 × 10^5^ steps. The resulting log files were analysed in Tracer v1.7.2 (Rambaut et al. 2018) to verify that the effective sample size for all parameters exceeded 200, following a conservative 10% burn-in. Finally, the maximum clade credibility tree was summarized using TreeAnnotator (https://beast.community/treeannotator) and visualized *via* FigTree v1.4.4 (http://tree.bio.ed.ac.uk/software/figtree/).

### 2.8. Statistical analysis

Student’s *t-*test were used to compare N/S ratios across groups. Student’s *t-*test was calculated with SciPy library (Virtanen et al. 2020). Statistical significance was defined as: ^*^P < .05, ^**^P < .01 and ^***^P < .001.

## 3. Results

### 3.1. Identification and characterization of five HAdV-C1 and C108 strains in Beijing

**Figure 1:**
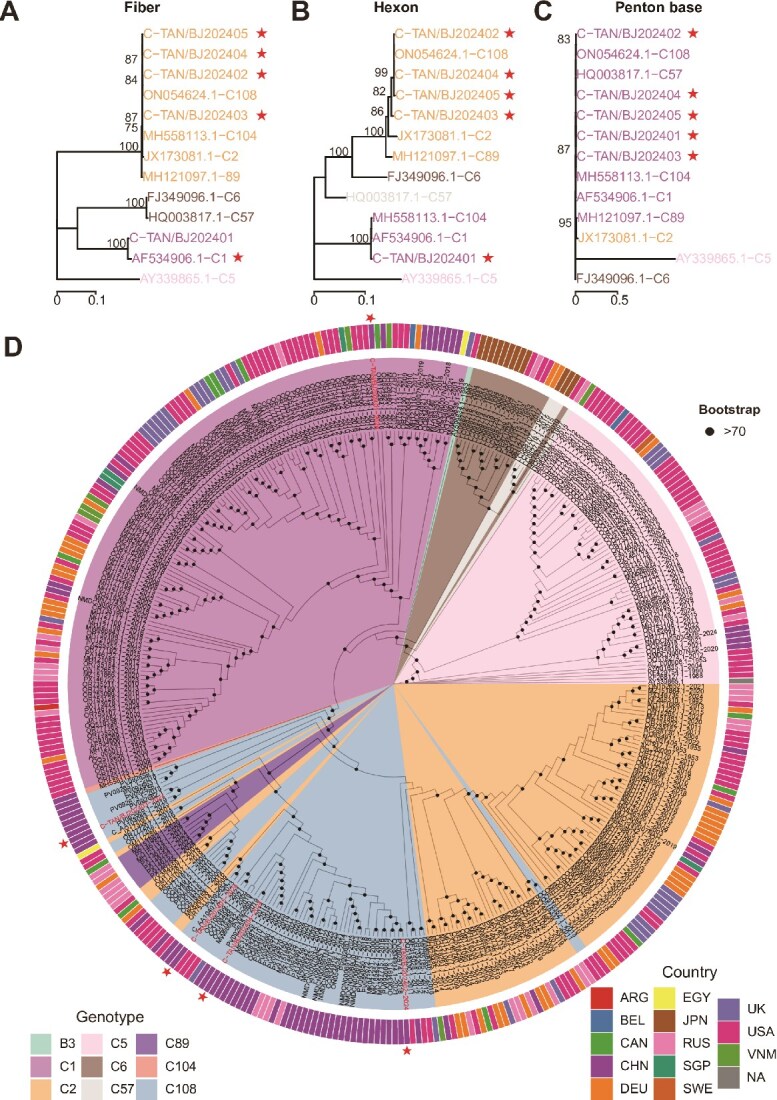
Phylogenetic tree based on the *fiber*, *hexon, penton base* and whole-genome sequence. The ML phylogenetic tree of *fiber* (A), *hexon* (B), *penton base* (C) and whole-genome sequence (D), including eight genotypes of HAdV-C1, HAdV-C2, HAdV-C5, HAdV-C6, HAdV-C57, HAdV-C89, HAdV-C104 and HAdV-C108. The five HAdV-C strains were highlighted in red colour and pentagrams. Colours in the outer ring represent countries, while the inner sectors are shown in different colours to represent genotypes.

WGS was conducted on clinical specimens obtained from patients with upper respiratory tract infections in Beijing between March 2023 and August 2024. We successfully identified a total of 26 HAdVs from 46 samples, including 21 genotype B (not included in this article) and 5 genotype C strains. The five HAdV-C strains were successfully characterized (Table 1), with genome lengths ranging from 35 933 to 35 998 bp. The average GC content was approximately 55.2%, and the mean genome coverage breadth reached 98%, aligning with the characteristic HAdV genome size of 34–36 kb. ML phylogenetic trees were constructed for the *penton base*, *hexon*, and *fiber* genes of the five HAdV-C strains identified in this study, incorporating eight reference prototype sequences. In the *fiber* tree, strains C-TAN/BJ202402–05 clustered with in a group comprising genotypes C2, C89, C104 and C108, whereas C-TAN/BJ202401 grouped with C1 (Fig. 1A). In the *hexon* tree, C-TAN/BJ202402–05 associated with C2, C89 and C108, while C-TAN/BJ202401 clustered with C1 and C104 (Fig. 1B). Regarding the *penton base* gene, all five strains (C-TAN/BJ202401–05) formed a monophyletic group with C57, C104 and C108 (Fig. 1C). In accordance with the HAdV Working Group classification criteria, C-TAN/BJ202401 was formally identified as type C1, while C-TAN/BJ202402–05 were assigned to type C108. These taxonomic assignments were further corroborated by pairwise nucleotide identity calculations across the *penton base, hexon* and *fiber* genes (Table S5). Furthermore, phylogenetic analysis based on whole genome sequences confirmed that C-TAN/BJ202401 clustered with type C1, while C-TAN/BJ202402–05 grouped with type C108 (Fig. 1D; Fig. S1). Notably, the four C108 strains exhibited discernible genetic divergence among themselves, suggesting ongoing intra-clade evolution or multiple introduction events within the region.

**Table 1:** Detailed information of the five HAdV-C strains of this study.

**Specimen ID**	**Gender**	**Age of patients**	**Clinical diagnosis**	**Collection date**	**P/H/F**
C-TAN/BJ202401	Female	11	Upper respiratory tract infection	2024/2/5	P1H1F1
C-TAN/BJ202402	Male	5	Upper respiratory tract infection	2024/6/24	P1H2F2
C-TAN/BJ202403	Female	10	Upper respiratory tract infection	2024/3/25	P1H2F2
C-TAN/BJ202404	Male	<1	Upper respiratory tract infection	2024/5/28	P1H2F2
C-TAN/BJ202405	Male	7	Upper respiratory tract infection	2024/5/28	P1H2F2

Note: ‘Age of patients’ refers solely to the patients’ chronological age at the time of data collection.

### 3.2. Multi-region recombination dynamics involving HAdV-C1, C5 and C6

**Figure 2:**
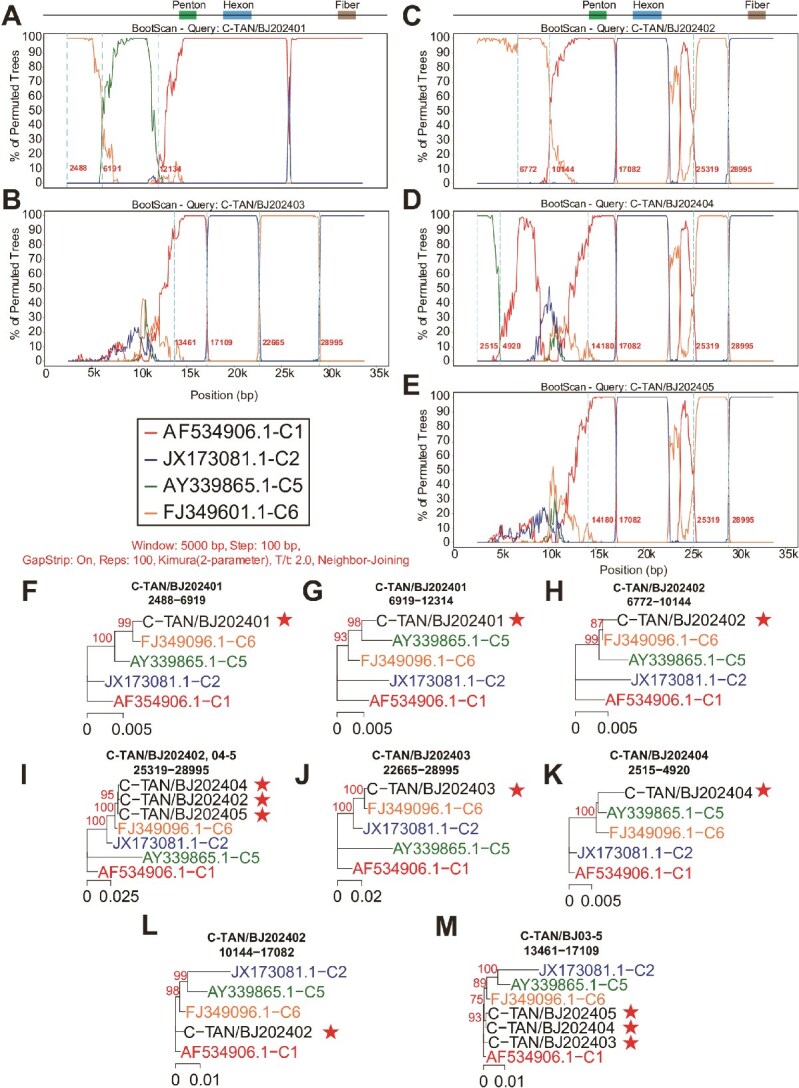
Recombination analysis of the five HAdV-C strains, a schematic diagram of the genome structure at the top of figures was displayed with the key regions labelled. Genomic recombination analyses performed with nine HAdV-C strains (A-E). The ML trees were constructed for eight identified recombination regions using sequences aligned with genotype strains of HAdV-C1, C2, C5 and C6. (F) C-TAN/BJ202401 (2488–6919 bp). (G) C-TAN/BJ202401 (6919–12 314 bp). (H) C-TAN/BJ202402 (6772–10 144 bp). (I) C-TAN/BJ202402 (10 144–17 082 bp). (J) C-TAN/BJ202403 (22665–28 995 bp). (K) C-TAN/BJ202404 (2515–4920 bp). (L) C-TAN/BJ202403–5 (13461–17 109 bp) (M) C-TAN/BJ202402,04–5 (25319–28 995 bp). The five HAdV-C strains were highlighted in pentagrams.

Recombination analysis of the whole-genome sequences revealed that all five HAdV-C strains identified in this study exhibited extensive genomic mosaicism. Specifically, strains C-TAN/BJ202401, 02 and 04 displayed unique recombinant architectures, harbouring discrete inter-typic segments derived from genotypes C5 and C6. These foreign segments were integrated at the 5′ terminus at coordinates 2488–6191 bp and 6191–12 314 bp (C6 and C5-derived in strain 01), 6772–10 144 bp (C6-derived in strain 02), 25 319–28 995 bp (C6-derived in strain 02, 04–5), 22 665–28 995 bp (C6-derived in strain 03),2515–4920 bp (C5-derived in strain 04), respectively (Figs. 2A, 2C–2D). These recombination events were further corroborated by segment-specific phylogenetic reconstruction, which demonstrated that these chimeric regions clustered with high nodal support with the C5 and C6 prototype strains (Fig. 2F-K). Moreover, three shared recombination patterns were identified among strains C-TAN/BJ202402–05. Specifically, inter-typic recombination events involving HAdV-C1-like segments were localized within two distinct genomic windows: (i) the 6 772–10 114 bp region of strains C-TAN/BJ202402 and (ii) the 13 461–17 109 bp region across strains C-TAN/BJ202403–05 (Fig. 2B–E). The validity of these mosaic patterns was further corroborated by segment-specific phylogenetic analysis, which displayed concordant topologies between the identified recombinant regions and the C1 prototype sequences (Fig. 2L-M).

Additionally, we assessed the statistical rigour of these recombination signals using a suite of detection algorithms. The results demonstrated that these events were robustly supported by multiple methods, consistently yielding high statistical significance (Table S6). In summary, we characterized six discrete recombination patterns across the five Beijing isolates, involving extensive genetic exchange among three parental lineages: HAdV-C1, C5 and C6.

### 3.3. Heterogeneous selective constraints across functional categories

**Figure 3:**
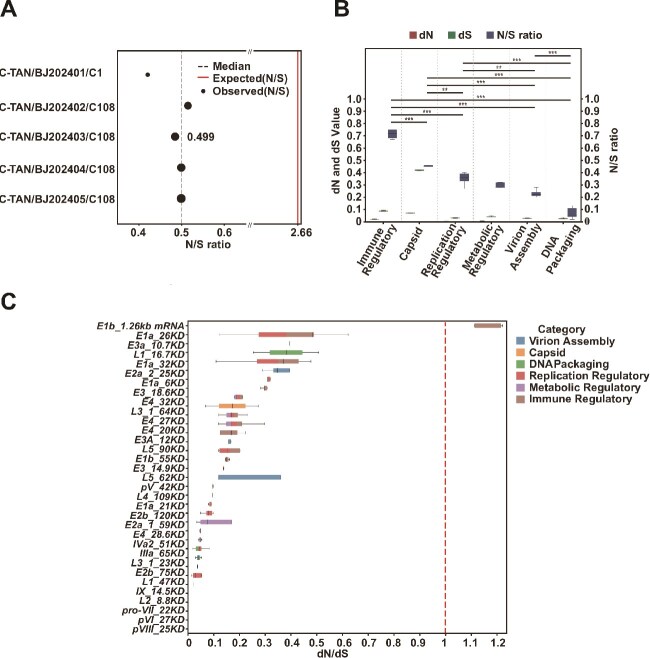
Selection pressure analysis of the five HAdV-C strains. This figure presented a comprehensive analysis of natural selection pressure on the five HAdV-C strains. By integrating genome-wide and gene-specific approaches, it quantified evolutionary constraints and highlighted functional variation in selective pressure across viral genes. (A) Distribution of observed non-synonymous to synonymous (N/S) substitution ratios across the whole genomes of the five HAdV-C strains. (B) Comparison of synonymous (dS) and non-synonymous (dN) substitution rates, along with corresponding N/S ratios, stratified by gene functional categories. (C) Gene-specific dN/dS ratios derived from pairwise comparisons between the clinical isolates and the HAdV-C1 prototype strain, illustrating functional variation in evolutionary pressure across the viral genome.

To elucidate the evolutionary forces shaping the HAdV-C genome, we analysed the ratio of non-synonymous to synonymous substitutions (N/S) across concatenated coding sequences. The five strains were concatenated and computationally compared with the reference sequence of C1 (AF534906.1). The observed genome-wide N/S ratios (median = 0.499) were significantly lower than the expected value (25653.7/9644.3 = 2.66), indicating a predominant regime of purifying selection (Fig. 3A). However, functional decomposition revealed substantial heterogeneity in selective constraints (Hou et al. 2022, Han et al. 2025). Viral genes were classified into the following functional categories: DNA packaging, virion assembly, immune regulation, replication regulation, metabolism regulation and capsid proteins (the *E2b_14KD* and *E1b_1.31Kb mRNA* genes were excluded due to unknown functions). Genes involved in immune regulation and capsid exhibited significantly larger N/S ratios compared to the highly conserved replication regulation genes (Student’s *t-*test, *P* < 0.01; Fig. 3B; Table S7). Notably, most genes remained under purifying selection (*dN/dS* < 1) (Fig. 3C).

### 3.4. Positive selection targets the disordered termini of E1A

**Figure 4:**
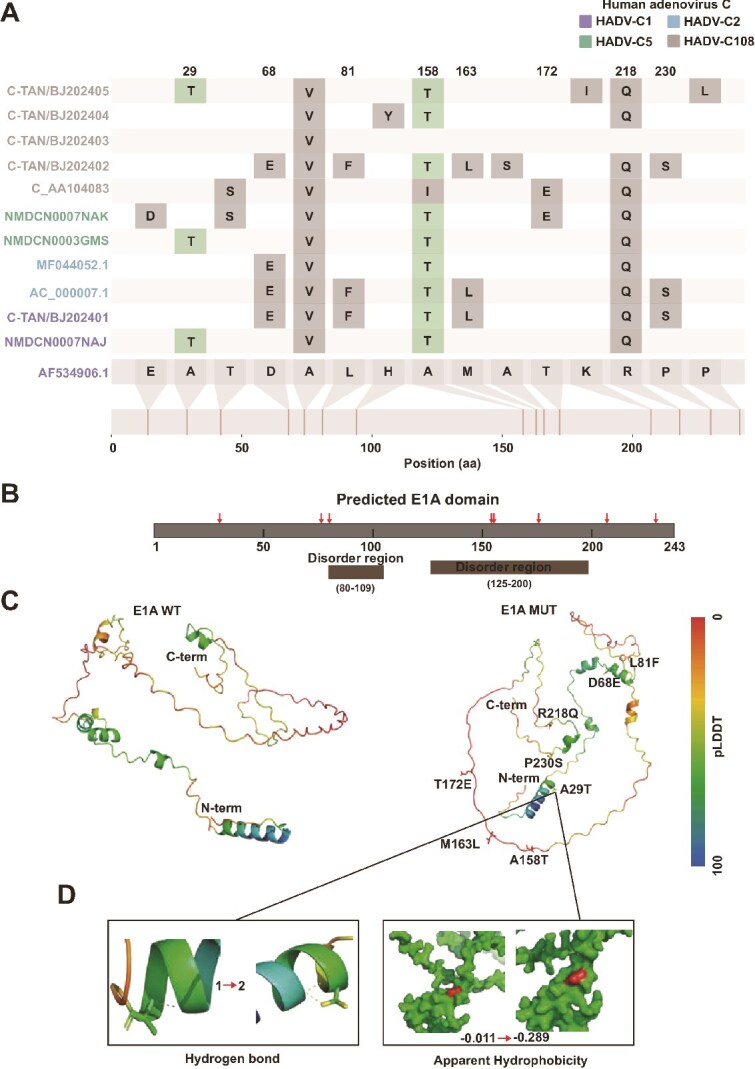
Identification and structural analysis of positive selection sites in the E1A protein (A) positive selection sites of the E1A proteins. Numbers above indicate specific positive selection sites. (B) Predicted domains of E1A by InterPro, with downward arrows denoting positive selection sites. (C) Wild-type E1A protein and substitution of E1A protein structure modelled using AlphaFold3 and visualized in PyMOL. Positive selection sites are numbered. The predicted structures in AlphaFold3 employ a rainbow colour scheme to represent pLDDT values. (D) Detailed analysis of the A29T substitution shows changes in hydrogen bonding and hydrophobicity, quantified and visualized in PyMOL (numerical values indicate differences in hydrogen bond counts and hydrophobicity).

Site-specific analysis identified positively selected residues in 23 genes, with a marked enrichment in immune-regulatory proteins, particularly E1A (Tables S8-S9). In the *E1a_26KD* gene, positive selection sites were preferentially localized to the N- and C-terminal regions (Fig. 4A-C), both of which are known to mediate immune-regulatory functions (Zemke and Berk 2017). Structural prediction using AlphaFold3 revealed that these variation hotspots map to intrinsically disordered regions (IDRs) rather than structured domains (Fig. 4B), underscoring the evolutionary plasticity of these regulatory segments. Structural modelling of the substitution of Alanine with Threonine at position 29 (A29T) substitution within the N-terminal IDR highlighted a potential mechanism for functional modulation. The A29T introduces an additional hydrogen bond (increasing from 1 to 2) and results in a drastic, ~ 30-fold increase in local surface hydrophilicity (ProtScale score: −0.011 to −0.289; Fig. 4D). Given that the E1A N-terminus is critical for inhibiting histone H2B ubiquitination and suppressing interferon-stimulated genes (Fonseca et al. 2012), such physicochemical alterations likely reshape the protein–protein interaction landscape required for immune evasion. Further investigation is needed to clarify the functional consequences of this substitution-induced shift in surface hydrophobicity.

### 3.5. Adaptive evolution of the flexible N-termini of E1B-55 K

**Figure 5:**
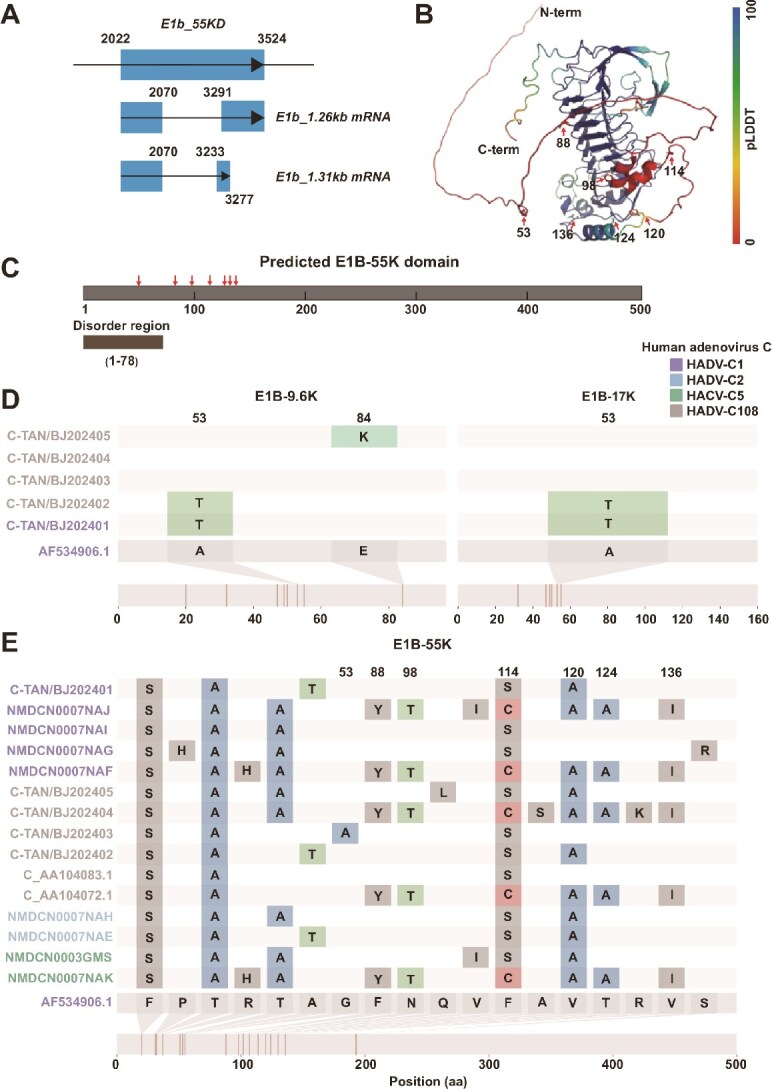
Positive selection sites of E1B-9.6 K, E1B-17 K, and E1B-55 K proteins. (A) Two splicing patterns of E1B-55 K. (B) Predicted three-dimensional structure of the E1B-55 K protein. Arrows and numbers indicate substitution sites. The colour bar represents pLDDT value. (C) Predicted domains of E1B-55 K. Arrows indicate substitution sites. (D) Positive selection sites of the E1B-9.6 K and E1B-17 K proteins. Numbers above indicate specific positive selection sites. (E) Sequences of E1B-55 K proteins from Chinese type C strains collected in this study. Numbers above indicate specific positive selection sites.

The *E1b_55KD* gene contains two splicing patterns that can encode the E1B-9.6 K and E1B-17 K proteins (Fig. 5A) (Sieber et al. 2011). The substitution multiples for these three proteins (E1B-9.6 K, E1B-17 K, and E1B-55 K) all rank within the top ten (E2B-14KD was excluded here), indicating strong positive selection (Tables S8-S9). Furthermore, we detected a common positive selection site at position 53 in the N-terminus of the E1B-55 K protein, which is also present in the other two proteins. Therefore, we focus our discussion on the selection sites within E1B-55 K. We identified seven positive selection sites within E1B-55 K (Fig. 5B; Tables S8-S9), most of which were clustered within the N-terminal IDR (Fig. 5B-C). Notably, the A53T substitution is conserved in the E1B-55 K isoform (Fig. 5D-E; Tables S8-S9), suggesting that shared evolutionary pressure exerts constraints on both proteins at this locus. The spatial distribution of these substitutions suggests a potential mechanism for fine-tuning nuclear trafficking and immune modulation. Specifically, the F88Y substitution is located adjacent to the nuclear export signal, while N98T and F114S lie in close proximity to the SUMO-conjugation motifs (Kolbe et al. 2022). Given that SUMOylation at residue K108 (The site corresponds to AF534906) regulates the nuclear export and p53-suppressive activity of E1B-55 K (Kolbe et al. 2022, Pennella et al. 2010, Berscheminski et al. 2016), these positively selected sites may modulate the efficiency of these critical regulatory domains.

## 4. Discussion

### 4.1. Lineage diversification of HAdV-C108

Phylogenomic reconstruction in our study categorized HAdV-C108 into five distinct clades, expanding upon the four-cluster classification proposed recently (Wang et al. 2025). While the prior study focused exclusively on C108 sequences, our inclusion of a broader range of HAdV-C genotypes provided the necessary evolutionary context to further resolve C108 diversification (Fig. 1D). Despite the identification of an additional clade, our results remain highly congruent with the four-cluster model, while further elucidating the complex ancestral relationships between C108 sub-lineages and other HAdV-C members. Specifically, strains C-TAN/BJ202402, 04 and 05 were assigned to Cluster 3, while C-TAN/BJ202403 clustered within Cluster 2. The absence of a novel, localized cluster suggests that Cluster 2 and Cluster 3 remain the predominant circulating lineages in China, reflecting a stable but diversifying population structure.

While HAdV-C briefly predominated in the post-NPI window, our genomic surveillance provides a high-resolution snapshot of its diversity. By reconstructing fine-scale maps, we identified six recombination events, including the stable P1H2F2 configuration (HAdV-C108). Although these specific recombination events likely represent historical genomic shifts rather than recent adaptations to NPIs, our findings highlight the complexity of HAdV-C evolution. The extent to which such recombination facilitates long-term circulation or periodic dominance remains an open question for future functional studies.

### 4.2. Heterogeneous selection pressure and convergent evolution in capsid proteins

The evolutionary success of HAdV-C is further explained by the heterogeneous selection pressures acting across its genome. While replication-essential genes remained under strong purifying selection to preserve fidelity, capsid proteins exhibited signs of adaptive evolution (Table 2). Notably, we identified a specific positive selection site, S458R, in penton base of the Chinese strains. Structural studies suggest that penton base residues in this region are critical for pentamer-pentamer interactions during the assembly of dodecahedral particles, which can mimic the viral entry process (Zubieta et al. 2005). The S458R substitution represents a striking example of convergent evolution, as it results in an amino acid substitution identical to that found in the previously dominant HAdV-B3 and B7 lineages at the corresponding position (Fig. S2) (Zubieta et al. 2005). We hypothesize that acquiring this ‘B-type-like’ feature may confer HAdV-C strains with enhanced stability or infection efficiency similar to HAdV-B, thereby providing a fitness advantage in the current population immune background. This molecular mimicry offers a plausible explanation for the changing epidemiological prevalence and underscores the potential of penton base as a target for broad-spectrum vaccine development (Zhou et al. 2025).

**Table 2:** Positive selection sites in *penton base*, *hexon* and *fiber*.

**Gene**	**Model compared**	**LRT *P*-value**	**Positive selection sites**
*Penton base*	M7 *versus* M8	0	157 K 1.000^**^, 420 A 0.997^**^, 458 S 0.998^**^
*Hexon*	M7 *versus* M8	0	No Data
*Fiber*	M7 *versus* M8	3.15 × 10^−5^	No Data

Note: a posterior probability > 0.99 (indicated by ^**^)

Our analysis of the E1B-55 K gene revealed that the sequences of NMDCN0007NAF/C1 and NMDCN0007NAK/C5 are completely identical (Fig. 5E), suggesting a potential recombination event between genotypes C1 and C5 in China. However, other type C sequences also exhibited a high degree of similarity (Fig. 5E). Therefore, whether the observed pattern in this gene within type C results from convergent evolution or recombination requires further investigation for confirmation.

### 4.3. Predicted adaptive fine-tuning of immunomodulatory proteins *via* IDR

Beyond structural proteins, our study provides novel insights into the putative adaptive evolution of the early regulatory proteins E1A and E1B-55 K. Unlike capsid substitutions that alter physical stability, positively selected sites in these regulatory proteins were predominantly enriched within IDRs (Sieber et al. 2011, Huang and Sarai 2012). For instance, the A29T substitution in the E1A N-terminus significantly alters local hydrophilicity (Fig. 5E), while the F88Y/N98T substitutions in E1B-55 K cluster near nuclear export and SUMOylation motifs. The flexibility of IDRs may allow these proteins to tolerate substitutions that could fine-tune interactions with host cellular machinery—such as inhibiting H2B ubiquitination or modulating p53 suppression—without disrupting overall protein stability (Huang and Sarai 2012). Intriguingly, protein homology alignment (Varadi et al. 2024) revealed significant similarity between the N-terminal region of E1B-55 K (residues 1–80) and the bacterial protein DUG3108 (Fig. S3A-B). The observation that position 53 is variable in both the viral protein and its bacterial homologue suggests that this residue serves as an evolutionarily conserved ‘hotspot’ for structural plasticity, potentially facilitating adaptation to diverse host environments. Together, these findings raise the possibility that HAdV-C may be undergoing a coordinated process of functional refinement alongside structural alteration, potentially enhancing its ability to evade host immune surveillance and facilitating sustained transmission within the human population. However, these interpretations remain speculative and require experimental validation.

### 4.4. Preliminary phylodynamic insights into the evolutionary histories of genotypes C1 and C108

We analysed the whole genomes of genotypes C1 and C108. The nucleotide substitution rate for C1 was estimated to be 1.856×10^−5^ mutations/site/year (95% HPD: 1.2273 × 10^−5^—2.9608 × 10^−^5), and the tMRCA was estimated to have occurred in 1430 (Fig. S4A). For C108, the nucleotide substitution rate was 2.89 × 10^−5^ mutations/site/year (95% HPD: 1.482 × 10^−5^—4.791×10^−5^), and the tMRCA of C-TAN/BJ202402–05 was estimated to be 2000, 1970, 2003 and 1945 respectively (Fig. S4B). The tMRCA indicates that these five sequences from Beijing did not emerge recently, and the specific details of their divergence warrant further investigation.

### 4.5. Limitations and future directions

Despite these insights, several limitations must be acknowledged. First, the modest sample size and restricted surveillance window may limit the generalizability of our findings, though the high diversity observed within this cohort implies a vast underlying recombinant reservoir. Second, the functional implications of the identified adaptive mutations (*e.g.* penton base: S458R and E1A: A29T) currently rely on *in silico* predictions. Future surveillance should prioritize: (i) expanding localized WGS databases to capture cryptic recombinant diversity, (ii) integrating wastewater-based epidemiology (Verani et al. 2024) with clinical sequencing to monitor community-level transmission and (iii) performing *in vitro* functional assays to validate the impact of IDR and capsid substitutions on viral fitness. Such a multi-faceted approach is essential to fully elucidate the evolutionary drivers of HAdV-C and inform the development of next-generation diagnostics and vaccines.

## 5. Conclusion

HAdV-C continues to represent a significant etiological threat to paediatric health, necessitating a profound understanding of its evolutionary mechanisms to bolster public health interventions. Based on a comprehensive genomic characterization of five HAdV-C strains isolated in Beijing (2023–2024), our findings elucidate that genomic recombination serves as a pivotal driver of lineage diversification, facilitating the emergence of complex mosaic architectures across the viral genome. Furthermore, the identification of heterogeneous selection pressures—particularly the adaptive mutations concentrated within the IDRs of immune-regulatory proteins—suggests a sophisticated ‘fine-tuning’ strategy employed by HAdV-C to circumvent host immune surveillance. Collectively, these observations delineate the contemporary evolutionary trajectories of HAdV-C in the post-NPI era and provide a critical genomic framework for the sustained surveillance, diagnostic development and control of emerging respiratory viral pathogens.

## Supplementary Material

Supplementary_File_for_review_veag040

## Data Availability

The sequence data generated in this study have been submitted to GenBase (C_AA134039.1-C_AA134043.1).

## References

[ref1] Abramson J, Adler J, Dunger J et al. Accurate structure prediction of biomolecular interactions with AlphaFold 3. Nature 2024;630:493–500. 10.1038/s41586-024-07487-w 38718835 PMC11168924

[ref2] Baele G, Ji X, Hassler GW et al. BEAST X for Bayesian phylogenetic, phylogeographic and phylodynamic inference. Nat Methods 2025;22:1653–6. 10.1038/s41592-025-02751-x 40624354 PMC12328226

[ref3] Bankevich A, Nurk S, Antipov D et al. SPAdes: a new genome assembly algorithm and its applications to single-cell sequencing. J Comput Biol 2012;19:455–77. 10.1089/cmb.2012.0021 22506599 PMC3342519

[ref4] Berscheminski J, Brun J, Speiseder T et al. Sp100A is a tumor suppressor that activates p53-dependent transcription and counteracts E1A/E1B-55K-mediated transformation. Oncogene 2016;35:3178–89. 10.1038/onc.2015.378 26477309

[ref5] Blum M, Andreeva A, Florentino LC et al. InterPro: the protein sequence classification resource in 2025. Nucleic Acids Res 2025;53:D444–56. 10.1093/nar/gkae1082 39565202 PMC11701551

[ref6] Camacho C, Coulouris G, Avagyan V et al. BLAST+: architecture and applications. BMC Bioinformatics 2009;10:421. https://www.ncbi.nlm.nih.gov/pubmed/20003500. 10.1186/1471-2105-10-421 20003500 PMC2803857

[ref7] Chen S, Zhou Y, Chen Y et al. Fastp: an ultra-fast all-in-one FASTQ preprocessor. Bioinformatics 2018;34:i884–90. 10.1093/bioinformatics/bty560 30423086 PMC6129281

[ref8] Dadkhah K, Dehghan S, Chodosh J et al. Evolution of human adenoviruses, a double-stranded DNA viral pathogen documented through genomics and bioinformatics and viewed through a web resource database. Viruses 2026;18:251. 10.3390/v18020251 41754594 PMC12944919

[ref9] Danecek P, Bonfield JK, Liddle J et al. Twelve years of SAMtools and BCFtools. Gigascience 2021;10:giab008. 10.1093/gigascience/giab008 33590861 PMC7931819

[ref10] Fonseca GJ, Thillainadesan G, Yousef AF et al. Adenovirus evasion of interferon-mediated innate immunity by direct antagonism of a cellular histone posttranslational modification. Cell Host Microbe 2012;11:597–606. 10.1016/j.chom.2012.05.005 22704620

[ref11] Gallardo J, Pérez-Illana M, Martín-González N et al. Adenovirus structure: what is new? Int J Mol Sci 2021;22:5240. 10.3390/ijms22105240 34063479 PMC8156859

[ref12] Gao F, Chen C, Arab DA et al. EasyCodeML: a visual tool for analysis of selection using CodeML. Ecol Evol 2019;9:3891–8. 10.1002/ece3.5015 31015974 PMC6467853

[ref13] Garrison E, Marth G. Haplotype-based variant detection from short-read sequencing. arXiv. 2012. 10.48550/arXiv.1207.3907

[ref14] Han X, Wu C, Deng Y et al. Decoding VZV's evolutionary arsenal: how Beijing strains use recombination and adaptive mutations to thrive. Virus Evol 2025;11:veaf076. 10.1093/ve/veaf076 41078487 PMC12513170

[ref15] Hou Y, Zhao S, Liu Q et al. Ongoing positive selection drives the evolution of SARS-CoV-2 genomes. Genomics Proteomics Bioinformatics 2022;20:1214–23. 10.1016/j.gpb.2022.05.009 35760317 PMC9233880

[ref16] Huang H, Sarai A. Analysis of the relationships between evolvability, thermodynamics, and the functions of intrinsically disordered proteins/regions. Comput Biol Chem 2012;41:51–7. 10.1016/j.compbiolchem.2012.10.001 23153654

[ref17] Ismail AM, Cui T, Dommaraju K et al. Genomic analysis of a large set of currently-and historically-important human adenovirus pathogens. Emerg Microbes Infect 2018;7:10. 10.1038/s41426-017-0004-y 29410402 PMC5837155

[ref18] Jianhua W, Lingjian Z, Yanhao H et al. Research progress on human adenovirus sepsis. Front Pediatr 2025;13:1552958. 10.3389/fped.2025.1552958 40458450 PMC12127347

[ref19] Jin R, Qin T, Li P et al. Increased circulation of adenovirus in China during 2023-2024: association with an increased prevalence of species B and school-associated transmission. J Inf Secur 2025;90:106475. 10.1016/j.jinf.2025.106475 40122244

[ref20] Kajon AE . Adenovirus infections: new insights for the clinical laboratory. J Clin Microbiol 2024;62:e0083622. 10.1128/jcm.00836-22 39189703 PMC11389149

[ref21] Katoh K, Standley DM. MAFFT multiple sequence alignment software version 7: improvements in performance and usability. Mol Biol Evol 2013;30:772–80. 10.1093/molbev/mst010 23329690 PMC3603318

[ref22] Kolbe V, Ip WH, Kieweg-Thompson L et al. Conserved E1B-55K SUMOylation in different human adenovirus species is a potent regulator of intracellular localization. J Virol 2022;96:e0083821. 10.1128/JVI.00838-21 34787461 PMC8826807

[ref23] Lei Y, Zhuang Z, Liu Y et al. Whole genomic sequence analysis of human adenovirus species C shows frequent recombination in Tianjin, China. Viruses 2023;15:1004. 10.3390/v15041004 37112985 PMC10142000

[ref24] Li H, Durbin R. Fast and accurate short read alignment with burrows-wheeler transform. Bioinformatics 2009;25:1754–60. 10.1093/bioinformatics/btp324 19451168 PMC2705234

[ref25] Li Z, Chen Q, Feng L et al. Active case finding with case management: the key to tackling the COVID-19 pandemic. Lancet 2020;396:63–70. 10.1016/S0140-6736(20)31278-2 32505220 PMC7272157

[ref26] Liang Y, Wei J, Shen J et al. Immunological pathogenesis and treatment progress of adenovirus pneumonia in children. Ital J Pediatr 2025;51:4. 10.1186/s13052-024-01836-1 39789604 PMC11715079

[ref27] Lole KS, Bollinger RC, Paranjape RS et al. Full-length human immunodeficiency virus type 1 genomes from subtype C-infected seroconverters in India, with evidence of intersubtype recombination. J Virol 1999;73:152–60. 10.1128/JVI.73.1.152-160.1999 9847317 PMC103818

[ref28] Lu J, Breitwieser FP, Thielen P et al. Bracken: estimating species abundance in metagenomics data. PeerJ Comput Sci 2017;3:e104. 10.7717/peerj-cs.104 PMC12016282 40271438

[ref29] Mah MG, Zeller MA, Zhang R et al. Discordant phylodynamic and spatiotemporal transmission patterns driving the long-term persistence and evolution of human coronaviruses. Npj Viruses 2024;2:49. 10.1038/s44298-024-00058-w 40295720 PMC11721344

[ref30] Mao NY, Zhu Z, Zhang Y et al. Current status of human adenovirus infection in China. World J Pediatr 2022;18:533–7. 10.1007/s12519-022-00568-8 35716276 PMC9206124

[ref31] Martin DP, Murrell B, Golden M et al. RDP4: detection and analysis of recombination patterns in virus genomes. Virus Evol 2015;1:vev003. 10.1093/ve/vev003 27774277 PMC5014473

[ref32] Minh BQ, Schmidt HA, Chernomor O et al. IQ-TREE 2: new models and efficient methods for phylogenetic inference in the genomic era. Mol Biol Evol 2020;37:1530–4. 10.1093/molbev/msaa015 32011700 PMC7182206

[ref33] Niu DD, Zhang Z, Chen ZG et al. The changed endemic pattern of human adenovirus from species C to B among children in 2022-2024 in Shenzhen, China. Sci Rep 2026;16:5902. 10.1038/s41598-026-36811-9 41565901 PMC12894712

[ref34] O'Toole A, Aziz A, Maloney D. Publication-ready single nucleotide polymorphism visualization with snipit. Bioinformatics 2024;40:btae510. 10.1093/bioinformatics/btae510 39137137 PMC11349183

[ref35] Pennella MA, Liu Y, Woo JL et al. Adenovirus E1B 55-kilodalton protein is a p53-SUMO1 E3 ligase that represses p53 and stimulates its nuclear export through interactions with promyelocytic leukemia nuclear bodies. J Virol 2010;84:12210–25. 10.1128/JVI.01442-10 20861261 PMC2976411

[ref36] Rambaut A, Lam TT, Max Carvalho L et al. Exploring the temporal structure of heterochronous sequences using TempEst (formerly path-O-gen). Virus Evol 2016;2:vew007. 10.1093/ve/vew007 27774300 PMC4989882

[ref37] Rambaut A, Drummond AJ, Xie D et al. Posterior summarization in Bayesian phylogenetics using tracer 1.7. Syst Biol 2018;67:901–4. 10.1093/sysbio/syy032 29718447 PMC6101584

[ref38] Robinson CM, Singh G, Lee JY et al. Molecular evolution of human adenoviruses. Sci Rep 2013;3:1812. 10.1038/srep01812 23657240 PMC3648800

[ref39] Russell WC . Adenoviruses: update on structure and function. J Gen Virol 2009;90:1–20. 10.1099/vir.0.003087-0 19088268

[ref40] Sanchez BC, Ortiz RM, Grasis JA. Human adenovirus serotype 5 infection dysregulates cysteine, purine, and unsaturated fatty acid metabolism in fibroblasts. FASEB J 2025;39:e70411. 10.1096/fj.202402726R 40052831 PMC11887610

[ref41] Sieber T, Scholz R, Spoerner M et al. Intrinsic disorder in the common N-terminus of human adenovirus 5 E1B-55K and its related E1BN proteins indicated by studies on E1B-93R. Virology 2011;418:133–43. 10.1016/j.virol.2011.07.012 21851959

[ref42] Varadi M, Bertoni D, Magana P et al. AlphaFold protein structure database in 2024: providing structure coverage for over 214 million protein sequences. Nucleic Acids Res 2024;52:D368–75. 10.1093/nar/gkad1011 37933859 PMC10767828

[ref43] Verani M, Pagani A, Federigi I et al. Wastewater-based epidemiology for viral surveillance from an endemic perspective: evidence and challenges. Viruses 2024;16:482. 10.3390/v16030482 38543847 PMC10975420

[ref44] Virtanen P, Gommers R, Oliphant TE et al. SciPy 1.0: fundamental algorithms for scientific computing in python. Nat Methods 2020;17:261–72. 10.1038/s41592-019-0686-2 32015543 PMC7056644

[ref45] Wang F, Zhu R, Qian Y et al. The changed endemic pattern of human adenovirus from species B to C among pediatric patients under the pressure of non-pharmaceutical interventions against COVID-19 in Beijing, China. Virol J 2023;20:4. 10.1186/s12985-023-01962-y 36624458 PMC9828375

[ref46] Wang F, De R, Han Z et al. High-frequency recombination of human adenovirus in children with acute respiratory tract infections in Beijing, China. Viruses 2024;16:828. 10.3390/v16060828 38932121 PMC11209268

[ref47] Wang J, Jing L, Duan Y et al. Genetic analysis of human adenovirus type 108 circulating in China during 2014-2024. Virol Sin 2025;40:694–709. 10.1016/j.virs.2025.09.002 40976392 PMC12665428

[ref48] Wood DE, Lu J, Langmead B. Improved metagenomic analysis with kraken 2. Genome Biol 2019;20:257. 10.1186/s13059-019-1891-0 31779668 PMC6883579

[ref49] Wu C, Zhang Y, Liang A et al. Comparative analysis between genotypes of adenovirus isolates from hospitalized children with acute respiratory tract infections and clinical manifestations in Wuhan, China, from June 2022 to September 2023. Virol Sin 2025;40:50–60. 10.1016/j.virs.2024.12.004 39710326 PMC11963011

[ref50] Yang Z . PAML 4: phylogenetic analysis by maximum likelihood. Mol Biol Evol 2007;24:1586–91. 10.1093/molbev/msm088 17483113

[ref51] Yu G, Lam TT, Zhu H et al. Two methods for mapping and visualizing associated data on phylogeny using Ggtree. Mol Biol Evol 2018;35:3041–3. 10.1093/molbev/msy194 30351396 PMC6278858

[ref52] Zang J, Xing Y, Zhang H et al. Molecular characteristics of human adenovirus isolated from the 2024 influenza-like illness outbreaks in Suzhou City, China. Virol J 2025;22:243. 10.1186/s12985-025-02870-z 40665385 PMC12265240

[ref53] Zemke NR, Berk AJ. The adenovirus E1A C terminus suppresses a delayed antiviral response and modulates RAS signaling. Cell Host Microbe 2017;22:789–800.e5. 10.1016/j.chom.2017.11.008 29241042 PMC5736016

[ref54] Zhang Z, Jiang S, Jiang H et al. Rapid genotype recognition of human adenovirus based on surface-enhanced Raman scattering combined with machine learning. Sensors Actuators B Chem 2024;400:134873. 10.1016/j.snb.2023.134873

[ref55] Zhao H, Liu Y, Feng Z et al. A fatal case of viral sepsis and encephalitis in a child caused by human adenovirus type 7 infection. Virol J 2022;19:154. 10.1186/s12985-022-01886-z 36171632 PMC9517974

[ref56] Zhou L, Yang Y, Mo C et al. Human adenovirus penton dodecahedron nanoparticles induce an enhanced neutralizing antibody response in mouse model. Vaccine 2025;61:127380. 10.1016/j.vaccine.2025.127380 40482460

[ref57] Zubieta C, Schoehn G, Chroboczek J et al. The structure of the human adenovirus 2 penton. Mol Cell 2005;17:121–35. 10.1016/j.molcel.2004.11.041 15629723

