# Supplementary material for: Genomic characterization and evolutionary dynamics of human adenovirus C (HAdV-C) in Beijing (2023–2024): insights into multiple recombination and adaptive evolution
Source: Virus Evol. 2026 Jul 1;12(1):veag040. doi: 10.1093/ve/veag040 (PMC13390923; doi:10.1093/ve/veag040)
Supplement: Supplementary_File_for_review_veag040 [file supplementary_file_for_review_veag040.docx]

**Table S1 The information of all the 375 sequences used in phylogenetic analysis**

| **Accession number** | **Country** | **Date** | **Genotype** | **Source** |
| --- | --- | --- | --- | --- |
| AY599834.1 | USA | 1953 | B3 | Genbank |
| AF534906.1 | USA | 1953 | C1 | Genbank |
| MH183293.1 | CHN | 2016 | C1 | Genbank |
| MN513345.1 | SGP | 2016 | C1 | Genbank |
| MN513341.1 | SGP | 2016 | C1 | Genbank |
| MN513344.1 | SGP | 2016 | C1 | Genbank |
| MN737436.1 | CHN | 2018 | C1 | Genbank |
| MW686821.1 | UK | 2017 | C1 | Genbank |
| MW686822.1 | UK | 2017 | C1 | Genbank |
| MW686823.1 | UK | 2017 | C1 | Genbank |
| MW686824.1 | UK | 2016 | C1 | Genbank |
| MW686827.1 | UK | 2017 | C1 | Genbank |
| MW686828.1 | UK | 2017 | C1 | Genbank |
| MW686831.1 | UK | 2017 | C1 | Genbank |
| MW686832.1 | UK | 2017 | C1 | Genbank |
| MW686833.1 | UK | 2018 | C1 | Genbank |
| MW686834.1 | UK | 2018 | C1 | Genbank |
| MW686838.1 | UK | 2019 | C1 | Genbank |
| MW686839.1 | UK | 2019 | C1 | Genbank |
| MW686843.1 | UK | 2018 | C1 | Genbank |
| MW686825.1 | UK | 2016 | C1 | Genbank |
| OM112288.1 | CAN | 2015 | C1 | Genbank |
| OM112290.1 | CAN | 2018 | C1 | Genbank |
| OM112292.1 | CAN | 2015 | C1 | Genbank |
| OM112293.1 | CAN | 2015 | C1 | Genbank |
| OM112294.1 | CAN | 2016 | C1 | Genbank |
| OQ518262.1 | USA | 2009 | C1 | Genbank |
| OQ518280.1 | USA | 2009 | C1 | Genbank |
| OQ518301.1 | USA | 2013 | C1 | Genbank |
| OQ518310.1 | USA | 2011 | C1 | Genbank |
| OQ518338.1 | USA | 2012 | C1 | Genbank |
| OQ518347.1 | USA | 2013 | C1 | Genbank |
| OQ518349.1 | USA | 2012 | C1 | Genbank |
| OQ518259.1 | USA | 2011 | C1 | Genbank |
| OQ518269.1 | USA | 2010 | C1 | Genbank |
| OQ518270.1 | USA | 1968 | C1 | Genbank |
| OQ518300.1 | USA | 2009 | C1 | Genbank |
| OQ518311.1 | USA | 2009 | C1 | Genbank |
| OQ518346.1 | USA | 2013 | C1 | Genbank |
| OR735182.1 | USA | 2012 | C1 | Genbank |
| OR735184.1 | USA | 2011 | C1 | Genbank |
| OR735189.1 | USA | 2011 | C1 | Genbank |
| OR735198.1 | USA | 2013 | C1 | Genbank |
| OR735200.1 | USA | 2012 | C1 | Genbank |
| OR735206.1 | USA | 2011 | C1 | Genbank |
| OR735211.1 | USA | 2010 | C1 | Genbank |
| OR735181.1 | USA | 2011 | C1 | Genbank |
| OR735190.1 | USA | 2012 | C1 | Genbank |
| OR735192.1 | USA | 2012 | C1 | Genbank |
| OR735194.1 | USA | 2011 | C1 | Genbank |
| OR735199.1 | USA | 2013 | C1 | Genbank |
| OR735203.1 | USA | 2009 | C1 | Genbank |
| OR735195.1 | USA | 2012 | C1 | Genbank |
| OR753096.1 | USA | 2010 | C1 | Genbank |
| OR753107.1 | USA | 2011 | C1 | Genbank |
| OR753111.1 | USA | 2009 | C1 | Genbank |
| OR753128.1 | USA | 2010 | C1 | Genbank |
| OR753130.1 | USA | 2009 | C1 | Genbank |
| OR753138.1 | USA | 2011 | C1 | Genbank |
| OR753112.1 | USA | 2011 | C1 | Genbank |
| OR753113.1 | USA | 2012 | C1 | Genbank |
| OR753115.1 | USA | 2011 | C1 | Genbank |
| OR753125.1 | USA | 2009 | C1 | Genbank |
| OR753129.1 | USA | 2010 | C1 | Genbank |
| OR753132.1 | USA | 2012 | C1 | Genbank |
| OR777164.1 | USA | 2019 | C1 | Genbank |
| OR777231.1 | USA | 2018 | C1 | Genbank |
| OR777163.1 | USA | 2019 | C1 | Genbank |
| OR777165.1 | USA | 2020 | C1 | Genbank |
| OR777172.1 | USA | 2010 | C1 | Genbank |
| OR777176.1 | USA | 2012 | C1 | Genbank |
| OR777177.1 | USA | 2013 | C1 | Genbank |
| OR777178.1 | USA | 2013 | C1 | Genbank |
| OR777192.1 | USA | 2015 | C1 | Genbank |
| OR777194.1 | USA | 2015 | C1 | Genbank |
| OR777214.1 | USA | 2017 | C1 | Genbank |
| OR777216.1 | USA | 2017 | C1 | Genbank |
| OR777221.1 | USA | 2017 | C1 | Genbank |
| OR777223.1 | USA | 2017 | C1 | Genbank |
| OR777225.1 | USA | 2017 | C1 | Genbank |
| OR777182.1 | USA | 2015 | C1 | Genbank |
| JX173082.1 | USA | 2003 | C1 | Genbank |
| JX173086.1 | USA | 2004 | C1 | Genbank |
| JX173083.1 | USA | 2003 | C1 | Genbank |
| JX173078.1 | ARG | 2000 | C1 | Genbank |
| JX173080.1 | EGY | 2001 | C1 | Genbank |
| PX146725.1 | RUS | 2023 | C1 | Genbank |
| PX146720.1 | RUS | 2023 | C1 | Genbank |
| PX146722.1 | RUS | 2023 | C1 | Genbank |
| PX146723.1 | RUS | 2024 | C1 | Genbank |
| PX255352.1 | RUS | 2023 | C1 | Genbank |
| AC_000017.1 | USA | 1953 | C1 | Genbank |
| NMDCN0007NAF | CHN | 2018 | C1 | NMDC |
| NMDCN0007NAG | CHN | 2020 | C1 | NMDC |
| NMDCN0007NAI | CHN | 2019 | C1 | NMDC |
| NMDCN0007NAJ | CHN | 2021 | C1 | NMDC |
| C-TAN/BJ202401 | CHN | 2024 | C1 | Genbase |
| MH121073.1 | DEU | 2002 | C1 | Genbank |
| MH121074.1 | DEU | 2002 | C1 | Genbank |
| MH121075.1 | DEU | 2008 | C1 | Genbank |
| MH121076.1 | DEU | 2008 | C1 | Genbank |
| MH121081.1 | DEU | 2012 | C1 | Genbank |
| MH121082.1 | DEU | 2012 | C1 | Genbank |
| MH121087.1 | DEU | 2013 | C1 | Genbank |
| MH121089.1 | DEU | 2013 | C1 | Genbank |
| MH121091.1 | DEU | 2014 | C1 | Genbank |
| MH121098.1 | DEU | 2015 | C1 | Genbank |
| MH121100.1 | DEU | 2015 | C1 | Genbank |
| MH121108.1 | DEU | 2016 | C1 | Genbank |
| MH121110.1 | DEU | 2017 | C1 | Genbank |
| MH121116.1 | DEU | 2017 | C1 | Genbank |
| MH121117.1 | DEU | 2017 | C1 | Genbank |
| MH828480.1 | VNM | 2014 | C1 | Genbank |
| MH828481.1 | VNM | 2014 | C1 | Genbank |
| MH828482.1 | VNM | 2015 | C1 | Genbank |
| MH828483.1 | VNM | 2015 | C1 | Genbank |
| MH828484.1 | VNM | 2014 | C1 | Genbank |
| MK805085.1 | CHN | 2009 | C1 | Genbank |
| MT263140.1 | CHN | 2017 | C1 | Genbank |
| MZ151860.1 | RUS | 2020 | C1 | Genbank |
| MZ151866.1 | RUS | 2020 | C1 | Genbank |
| MZ603084.1 | BEL | 2010 | C1 | Genbank |
| ON152653.1 | RUS | 2021 | C1 | Genbank |
| OQ834920.1 | CHN | 2021 | C1 | Genbank |
| OQ834921.1 | CHN | 2021 | C1 | Genbank |
| OQ834922.1 | CHN | 2021 | C1 | Genbank |
| OQ834923.1 | CHN | 2021 | C1 | Genbank |
| OQ834925.1 | CHN | 2022 | C1 | Genbank |
| J01917.1 | USA | 1953 | C2 | Genbank |
| NC_001405.1 | USA | 1955 | C2 | Genbank |
| PX146729.1 | RUS | 2023 | C2 | Genbank |
| PX146742.1 | RUS | 2023 | C2 | Genbank |
| PX146735.1 | RUS | 2024 | C2 | Genbank |
| PX146736.1 | RUS | 2024 | C2 | Genbank |
| PX146732.1 | RUS | 2024 | C2 | Genbank |
| PX146740.1 | RUS | 2024 | C2 | Genbank |
| MF044052.1 | CHN | 2014 | C2 | Genbank |
| KX384959.1 | USA | 2002 | C2 | Genbank |
| KF268130.1 | USA | 2004 | C2 | Genbank |
| KF268310.1 | USA | 1992 | C2 | Genbank |
| MN513342.1 | SGP | 2016 | C2 | Genbank |
| MW686811.1 | UK | 2015 | C2 | Genbank |
| MW686813.1 | UK | 2015 | C2 | Genbank |
| MW686815.1 | UK | 2015 | C2 | Genbank |
| MW686817.1 | UK | 2015 | C2 | Genbank |
| MW686830.1 | UK | 2018 | C2 | Genbank |
| MW686836.1 | UK | 2019 | C2 | Genbank |
| MW686837.1 | UK | 2019 | C2 | Genbank |
| MW686850.1 | UK | 2018 | C2 | Genbank |
| MW686812.1 | UK | 2015 | C2 | Genbank |
| MW686829.1 | UK | 2018 | C2 | Genbank |
| MW686841.1 | UK | 2019 | C2 | Genbank |
| OQ518271.1 | USA | 2011 | C2 | Genbank |
| OQ518290.1 | USA | 2009 | C2 | Genbank |
| OQ518312.1 | USA | 2010 | C2 | Genbank |
| OQ518325.1 | USA | 2009 | C2 | Genbank |
| OQ518261.1 | USA | 2011 | C2 | Genbank |
| OQ518272.1 | USA | 2009 | C2 | Genbank |
| OQ518285.1 | USA | 2010 | C2 | Genbank |
| OQ518309.1 | USA | 2012 | C2 | Genbank |
| OQ518335.1 | USA | 2010 | C2 | Genbank |
| OQ518350.1 | USA | 2010 | C2 | Genbank |
| OR735187.1 | USA | 2011 | C2 | Genbank |
| OR735202.1 | USA | 2011 | C2 | Genbank |
| OR735196.1 | USA | 2013 | C2 | Genbank |
| OR735186.1 | USA | 2010 | C2 | Genbank |
| OR753114.1 | USA | 2009 | C2 | Genbank |
| OR753117.1 | USA | 2010 | C2 | Genbank |
| OR777170.1 | USA | 2010 | C2 | Genbank |
| OR777213.1 | USA | 2016 | C2 | Genbank |
| OR777220.1 | USA | 2017 | C2 | Genbank |
| OR777167.1 | USA | 2010 | C2 | Genbank |
| PP068615.1 | USA | 2010 | C2 | Genbank |
| PP079214.1 | USA | 2012 | C2 | Genbank |
| JX173084.1 | USA | 2003 | C2 | Genbank |
| JX173081.1 | EGY | 2001 | C2 | Genbank |
| PX146728.1 | RUS | 2024 | C2 | Genbank |
| AC_000007.1 | USA | 1953 | C2 | Genbank |
| NMDCN0007NAH | CHN | 2019 | C2 | NMDC |
| MH121097.1 | DEU | 2019 | C2 | Genbank |
| MH121070.1 | DEU | 2000 | C2 | Genbank |
| MH121071.1 | DEU | 2002 | C2 | Genbank |
| MH121072.1 | DEU | 2000 | C2 | Genbank |
| MH121077.1 | DEU | 2009 | C2 | Genbank |
| MH121080.1 | DEU | 2012 | C2 | Genbank |
| MH121083.1 | DEU | 2012 | C2 | Genbank |
| MH121084.1 | DEU | 2012 | C2 | Genbank |
| MH121085.1 | DEU | 2012 | C2 | Genbank |
| MH121086.1 | DEU | 2013 | C2 | Genbank |
| MH121088.1 | DEU | 2013 | C2 | Genbank |
| MH121090.1 | DEU | 2014 | C2 | Genbank |
| MH121093.1 | DEU | 2014 | C2 | Genbank |
| MH121096.1 | DEU | 2015 | C2 | Genbank |
| MH121101.1 | DEU | 2015 | C2 | Genbank |
| MH121102.1 | DEU | 2015 | C2 | Genbank |
| MH121103.1 | DEU | 2015 | C2 | Genbank |
| MH121104.1 | DEU | 2015 | C2 | Genbank |
| MH121105.1 | DEU | 2015 | C2 | Genbank |
| MH121106.1 | DEU | 2016 | C2 | Genbank |
| MH121107.1 | DEU | 2016 | C2 | Genbank |
| MH121109.1 | DEU | 2017 | C2 | Genbank |
| MH121111.1 | DEU | 2017 | C2 | Genbank |
| MH121115.1 | DEU | 2017 | C2 | Genbank |
| MH828485.1 | VNM | 2014 | C2 | Genbank |
| MK836309.1 | CHN | 2009 | C2 | Genbank |
| MK896858.1 | CHN | 2016 | C2 | Genbank |
| MZ151861.1 | RUS | 2019 | C2 | Genbank |
| MZ151863.1 | RUS | 2020 | C2 | Genbank |
| MZ151864.1 | RUS | 2020 | C2 | Genbank |
| OM112284.1 | CAN | 2016 | C2 | Genbank |
| OM112285.1 | CAN | 2015 | C2 | Genbank |
| OM112286.1 | CAN | 2018 | C2 | Genbank |
| OM112287.1 | CAN | 2018 | C2 | Genbank |
| OM112289.1 | CAN | 2015 | C2 | Genbank |
| OM112291.1 | CAN | 2015 | C2 | Genbank |
| ON152651.1 | RUS | 2021 | C2 | Genbank |
| ON152652.1 | RUS | 2021 | C2 | Genbank |
| OQ834911.1 | CHN | 2022 | C2 | Genbank |
| OQ834912.1 | CHN | 2022 | C2 | Genbank |
| AY339865.1 |  |  | C5 | Genbank |
| AY601635.1 | USA | 2004 | C5 | Genbank |
| MW686844.1 | UK | 2018 | C5 | Genbank |
| MW686846.1 | UK | 2018 | C5 | Genbank |
| MW686847.1 | UK | 2018 | C5 | Genbank |
| MW686848.1 | UK | 2019 | C5 | Genbank |
| MW686849.1 | UK | 2019 | C5 | Genbank |
| MW686845.1 | UK | 2019 | C5 | Genbank |
| OQ518268.1 | USA | 2011 | C5 | Genbank |
| OQ518274.1 | USA | 2012 | C5 | Genbank |
| OQ518283.1 | USA | 2009 | C5 | Genbank |
| OQ518303.1 | USA | 2011 | C5 | Genbank |
| OQ518305.1 | USA | 2010 | C5 | Genbank |
| OQ518307.1 | USA | 2012 | C5 | Genbank |
| OQ518263.1 | USA | 2012 | C5 | Genbank |
| OQ518275.1 | USA | 2010 | C5 | Genbank |
| OQ518277.1 | USA | 2011 | C5 | Genbank |
| OQ518289.1 | USA | 2012 | C5 | Genbank |
| OQ518296.1 | USA | 2012 | C5 | Genbank |
| OQ518319.1 | USA | 2009 | C5 | Genbank |
| OQ518340.1 | USA | 2009 | C5 | Genbank |
| OQ518353.1 | USA | 2012 | C5 | Genbank |
| OR735183.1 | USA | 2009 | C5 | Genbank |
| OR735188.1 | USA | 2011 | C5 | Genbank |
| OR735179.1 | USA | 2011 | C5 | Genbank |
| OR728260.1 | USA | 2021 | C5 | Genbank |
| OR753099.1 | USA | 2011 | C5 | Genbank |
| OR753108.1 | USA | 2011 | C5 | Genbank |
| OR777173.1 | USA | 2018 | C5 | Genbank |
| OR777181.1 | USA | 2015 | C5 | Genbank |
| OR777218.1 | USA | 2017 | C5 | Genbank |
| OR777154.1 | USA | 2020 | C5 | Genbank |
| OR777157.1 | USA | 2019 | C5 | Genbank |
| OR777174.1 | USA | 2019 | C5 | Genbank |
| OR777190.1 | USA | 2015 | C5 | Genbank |
| OR777205.1 | USA | 2016 | C5 | Genbank |
| OR777217.1 | USA | 2017 | C5 | Genbank |
| OR777241.1 | USA | 2019 | C5 | Genbank |
| OR777232.1 | USA | 2018 | C5 | Genbank |
| OR876398.1 | USA | 2010 | C5 | Genbank |
| PP070539.1 | USA | 2011 | C5 | Genbank |
| KF429754.1 | USA | 1990 | C5 | Genbank |
| KF268127.1 | USA | 1988 | C5 | Genbank |
| KX868466.2 | SWE | 2000 | C5 | Genbank |
| PX146747.1 | RUS | 2024 | C5 | Genbank |
| PX146748.1 | RUS | 2024 | C5 | Genbank |
| PX146744.1 | RUS | 2024 | C5 | Genbank |
| AC_000008.1 | USA | 1953 | C5 | Genbank |
| NMDCN0007NAK | CHN | 2020 | C5 | NMDC |
| NMDCN0003GMS | CHN | 2024 | C5 | NMDC |
| MH121094.1 | DEU | 2014 | C5 | Genbank |
| MH121118.1 | DEU | 2015 | C5 | Genbank |
| MH121119.1 | DEU | 2017 | C5 | Genbank |
| MH828486.1 | VNM | 2015 | C5 | Genbank |
| MZ603080.1 | BEL | 2008 | C5 | Genbank |
| ON152649.1 | RUS | 2022 | C5 | Genbank |
| ON152650.1 | RUS | 2022 | C5 | Genbank |
| OQ834916.1 | CHN | 2021 | C5 | Genbank |
| OQ834918.1 | CHN | 2021 | C5 | Genbank |
| LC068720.1 | JPN | 2005 | C6 | Genbank |
| LC068719.1 | JPN | 2005 | C6 | Genbank |
| LC068718.1 | JPN | 2004 | C6 | Genbank |
| LC068717.1 | JPN | 2004 | C6 | Genbank |
| LC068716.1 | JPN | 2003 | C6 | Genbank |
| LC068715.1 | JPN | 1994 | C6 | Genbank |
| LC068714.1 | JPN | 1993 | C6 | Genbank |
| LC068713.1 | JPN | 1987 | C6 | Genbank |
| LC068712.1 | JPN | 1987 | C6 | Genbank |
| FJ349096.1 | USA | 1953 | C6 | Genbank |
| HQ413315.1 | USA | 1953 | C6 | Genbank |
| OP871032.1 | RUS | 2011 | C6 | Genbank |
| JX423389.1 | USA | 2007 | C6 | Genbank |
| OR753103.1 | USA | 2010 | C6 | Genbank |
| MH121112.1 | DEU | 2017 | C6 | Genbank |
| MH121113.1 | DEU | 2017 | C6 | Genbank |
| HQ003817.1 | RUS | 1997 | C57 | Genbank |
| LC062716.1 | JPN | 2015 | C57 | Genbank |
| LC062717.1 | JPN | 2015 | C57 | Genbank |
| LC068707.1 | JPN | 1997 | C57 | Genbank |
| PX146753.1 | RUS | 2023 | C89 | Genbank |
| PX146754.1 | RUS | 2024 | C89 | Genbank |
| PX146755.1 | RUS | 2024 | C89 | Genbank |
| PX146756.1 | RUS | 2024 | C89 | Genbank |
| PX146752.1 | RUS | 2024 | C89 | Genbank |
| MH121114.1 | DEU | 2017 | C89 | Genbank |
| MZ151862.1 | RUS | 2020 | C89 | Genbank |
| MH558113.1 | CHN | 2017 | C104 | Genbank |
| C-TAN/BJ202402 | CHN | 2024 | C108 | Genbase |
| C-TAN/BJ202403 | CHN | 2024 | C108 | Genbase |
| C-TAN/BJ202404 | CHN | 2024 | C108 | Genbase |
| C-TAN/BJ202405 | CHN | 2024 | C108 | Genbase |
| NMDCN0007NAE | CHN | 2017 | C108 | NMDC |
| ON054624.1 | CHN | 2014 | C108 | Genbank |
| MZ151865.1 | RUS | 2020 | C108 | Genbank |
| PX146759.1 | RUS | 2023 | C108 | Genbank |
| PX146760.1 | RUS | 2023 | C108 | Genbank |
| OQ108498.1 | USA | 2021 | C108 | Genbank |
| PX146758.1 | RUS | 2024 | C108 | Genbank |
| C_AA104083.1 | CHN | 2023 | C108 | Genbase |
| C_AA104072.1 | CHN | 2023 | C108 | Genbase |
| MK165452.1 | CHN | 2000 | C108 | Genbank |
| MW686840.1 | UK | 2018 | C108 | Genbank |
| PV092674.1 | CHN | 2018 | C108 | Genbank |
| PV092673.1 | CHN | 2018 | C108 | Genbank |
| PV092672.1 | CHN | 2018 | C108 | Genbank |
| PV092671.1 | CHN | 2018 | C108 | Genbank |
| PV092670.1 | CHN | 2017 | C108 | Genbank |
| PV092669.1 | CHN | 2019 | C108 | Genbank |
| PV092668.1 | CHN | 2019 | C108 | Genbank |
| PV092667.1 | CHN | 2020 | C108 | Genbank |
| PV092666.1 | CHN | 2021 | C108 | Genbank |
| PV092665.1 | CHN | 2022 | C108 | Genbank |
| PV092664.1 | CHN | 2023 | C108 | Genbank |
| PV092663.1 | CHN | ND | C108 | Genbank |
| PV092662.1 | CHN | ND | C108 | Genbank |
| PV092661.1 | CHN | ND | C108 | Genbank |
| PV092660.1 | CHN | ND | C108 | Genbank |
| PV092659.1 | CHN | ND | C108 | Genbank |
| PV092658.1 | CHN | ND | C108 | Genbank |
| PV092657.1 | CHN | 2018 | C108 | Genbank |
| PV092656.1 | CHN | 2018 | C108 | Genbank |
| PV092655.1 | CHN | 2019 | C108 | Genbank |
| PV092654.1 | CHN | ND | C108 | Genbank |
| OQ518339.1 | USA | 2009 | C108 | Genbank |
| OR777161.1 | USA | 2009 | C108 | Genbank |
| OQ518326.1 | USA | 2009 | C108 | Genbank |
| OQ518342.1 | USA | 2010 | C108 | Genbank |
| OQ518337.1 | USA | 2011 | C108 | Genbank |
| OQ518330.1 | USA | 2011 | C108 | Genbank |
| OQ518318.1 | USA | 2011 | C108 | Genbank |
| OR735185.1 | USA | 2012 | C108 | Genbank |
| OR735209.1 | USA | 2012 | C108 | Genbank |
| PP068613.1 | USA | 2013 | C108 | Genbank |
| OQ518352.1 | USA | 2013 | C108 | Genbank |
| OR777207.1 | USA | 2016 | C108 | Genbank |
| OR777208.1 | USA | 2016 | C108 | Genbank |
| OQ108499.1 | USA | 2021 | C108 | Genbank |
| MK041231.1 | CHN | 2000 | C108 | Genbank |
| MK165453.1 | CHN | 2004 | C108 | Genbank |
| KR699642.1 | CHN | 2009 | C108 | Genbank |
| MF315028.1 | CHN | 2012 | C108 | Genbank |
| MF315029.1 | CHN | 2013 | C108 | Genbank |
| MT424875.1 | CHN | 2015 | C108 | Genbank |
| MK357715.1 | CHN | 2016 | C108 | Genbank |
| OQ834919.1 | CHN | 2021 | C108 | Genbank |
| OQ834914.1 | CHN | 2021 | C108 | Genbank |
| OQ834915.1 | CHN | 2021 | C108 | Genbank |
| OQ834917.1 | CHN | 2021 | C108 | Genbank |
| OQ834910.1 | CHN | 2022 | C108 | Genbank |
| OQ834913.1 | CHN | 2022 | C108 | Genbank |
| MW686842.1 | UK | 2019 | C108 | Genbank |
| NMDCN00069U0 | CHN | 2023 | C108 | NMDC |
| NMDCN00069TR | CHN | 2023 | C108 | NMDC |
| NMDCN00069TO | CHN | 2023 | C108 | NMDC |
| NMDCN00069TM | CHN | 2023 | C108 | NMDC |
| NMDCN00069TL | CHN | 2023 | C108 | NMDC |

Notes:

Genbank: National Center for Biotechnology Information

Genbase: China national Center for Bioinformation

NMDC: National Microbial Data Center

ND: No Data

**Table S2 The information of 66 sequences used in selection analysis**

| **Accession number** | **Country** | **Date** | **Genotype** | **Source** |  |
| --- | --- | --- | --- | --- | --- |
| OR753138.1 | USA | 2011 | C1 | Genbank |  |
| OR753096.1 | USA | 2010 | C1 | Genbank |  |
| OR735199.1 | USA | 2013 | C1 | Genbank |  |
| OR777163.1 | USA | 2019 | C1 | Genbank |  |
| OR753130.1 | USA | 2009 | C1 | Genbank |  |
| C-TAN/BJ202401 | CHN | 2024 | C1 | Genbase |  |
| MW686834.1 | UK | 2018 | C1 | Genbank |  |
| OR753107.1 | USA | 2011 | C1 | Genbank |  |
| NMDCN0007NAI | CHN | 2019 | C1 | NMDC |  |
| OR753115.1 | USA | 2011 | C1 | Genbank |  |
| OR777192.1 | USA | 2015 | C1 | Genbank |  |
| MN513341.1 | SGP | 2016 | C1 | Genbank |  |
| OQ518300.1 | USA | 2009 | C1 | Genbank |  |
| OR753113.1 | USA | 2012 | C1 | Genbank |  |
| OQ518310.1 | USA | 2011 | C1 | Genbank |  |
| MN513344.1 | SGP | 2016 | C1 | Genbank |  |
| OR777182.1 | USA | 2015 | C1 | Genbank |  |
| OR777176.1 | USA | 2012 | C1 | Genbank |  |
| OM112290.1 | CAN | 2018 | C1 | Genbank |  |
| OM112294.1 | CAN | 2016 | C1 | Genbank |  |
| NMDCN0007NAF | CHN | 2018 | C1 | NMDC |  |
| NMDCN0007NAJ | CHN | 2021 | C1 | NMDC |  |
| MH183293.1 | CHN | 2016 | C1 | Genbank |  |
| OQ518347.1 | USA | 2013 | C1 | Genbank |  |
| OQ518311.1 | USA | 2009 | C1 | Genbank |  |
| OR777177.1 | USA | 2013 | C1 | Genbank |  |
| OR735184.1 | USA | 2011 | C1 | Genbank |  |
| OQ518338.1 | USA | 2012 | C1 | Genbank |  |
| MW686838.1 | UK | 2019 | C1 | Genbank |  |
| OR735206.1 | USA | 2011 | C1 | Genbank |  |
| NMDCN0007NAG | CHN | 2020 | C1 | NMDC |  |
| OR777220.1 | USA | 2017 | C2 | Genbank |  |
| MW686840.1 | UK | 2018 | C2 | Genbank |  |
| KX384959.1 | USA | 2002 | C2 | Genbank |  |
| OQ518272.1 | USA | 2009 | C2 | Genbank |  |
| OR735196.1 | USA | 2013 | C2 | Genbank |  |
| OR735186.1 | USA | 2010 | C2 | Genbank |  |
| OQ518261.1 | USA | 2011 | C2 | Genbank |  |
| AC_000007.1 | USA | 1953 | C2 | Genbank |  |
| MF044052.1 | CHN | 2014 | C2 | Genbank |  |
| OR753114.1 | USA | 2009 | C2 | Genbank |  |
| MN513342.1 | SGP | 2016 | C2 | Genbank |  |
| OR728260.1 | USA | 2021 | C5 | Genbank |  |
| AC_000008.1 | USA | 1953 | C5 | Genbank |  |
| NMDCN0007NAK | CHN | 2020 | C5 | NMDC |  |
| KX868466.2 | SWE | 2000 | C5 | Genbank |  |
| OQ518277.1 | USA | 2011 | C5 | Genbank |  |
| MW686848.1 | UK | 2019 | C5 | Genbank |  |
| NMDCN0003GMS | CHN | 2024 | C5 | NMDC |  |
| OR753099.1 | USA | 2011 | C5 | Genbank |  |
| OR777173.1 | USA | 2018 | C5 | Genbank |  |
| OR735179.1 | USA | 2011 | C5 | Genbank |  |
| OR777154.1 | USA | 2020 | C5 | Genbank |  |
| OQ518274.1 | USA | 2012 | C5 | Genbank |  |
| OR753108.1 | USA | 2011 | C5 | Genbank |  |
| OR753103.1 | USA | 2010 | C6 | Genbank |  |
| LC068715.1 | JPN | 1994 | C6 | Genbank |  |
| FJ349096.1 | USA | 1953 | C6 | Genbank |  |
| LC062717.1 | JPN | 2015 | C57 | Genbank |  |
| LC068707.1 | JPN | 1997 | C57 | Genbank |  |
| C-TAN/BJ202405 | CHN | 2024 | C108 | Genbase |  |
| C-TAN/BJ202402 | CHN | 2024 | C108 | Genbase |  |
| C-TAN/BJ202403 | CHN | 2024 | C108 | Genbase |  |
| C-TAN/BJ202404 | | CHN | 2024 | C108 | Genbase |
| C_AA104083.1 | CHN | 2023 | C108 | Genbase |  |
| C_AA104072.1 | CHN | 2023 | C108 | Genbase |  |

Notes:

Genbank: National Center for Biotechnology Information

Genbase: China national Center for Bioinformation

NMDC: National Microbial Data Center

**Table S3 The output of BF in C1 and C108**

| **C1** | | | **C108** | | |
| --- | --- | --- | --- | --- | --- |
| **Strict**  **Clock lnL (***H_0_*_:_**)** | **Uncorrelated Relaxed Clock lnL (***H_1_***)** | **2 ln(BF)** | **Strict Clock lnL (***H_0_*_:_**)** | **Uncorrelated Relaxed Clock lnL (***H_1_***)** | **2 ln(BF)** |
| -82459.37 | -82312.59 | 293.56 | -69448.99 | -69321.81 | 254.35 |

Notes：

BF: Bayes Factor

*H_0_*_:_ Strict Clock

*H_1_*_:_ Uncorrelated Relaxed Clock

To evaluate the fit of different molecular clock models for the C1 and C108 datasets, we performed Marginal Likelihood Estimation (MLE) using stepping-stone sampling (SS). The competing models—*H_0_* and *H_1_* —were compared using the BF (Baele et al. 2012).

**Table S4 Judgment Criteria(Kass and Raftery 1995)**

| **2ln (BF)** | **BF** | **Evidence against *H_0_*** |
| --- | --- | --- |
| 0 to 2 | 1 to 3 | Not worth more than a bare mention |
| 2 to 6 | 3 to 20 | Positive |
| 6 to 10 | 20 to 150 | Strong |
| > 10 | > 150 | Very Strong |

Note: This table is used as the standard for judging the strength of Bayes factors.

**Table S5 The nucleotide similarity of five HAdV-C with eight genotypes of HAdV-C in *fiber*, *hexon* and *penton base***

| **Accession number** | **AF534906.1-C1** | **JX173081.1-C2** | **AY339865.1-C5** | **FJ349096.1-C6** | **HQ003817.1-C57** | **MH121097.1-C89** | **MH558113.1-C104** | **ON054624.1-C108** | **F/H/P** |
| --- | --- | --- | --- | --- | --- | --- | --- | --- | --- |
| C-TAN/BJ202401 | 98.86% | 72.17% | 74.71% | 71.10% | 70.99% | 72.35% | 72.35% | 72.41% | F |
| C-TAN/BJ202402 | 72.24% | 99.43% | 72.54% | 67.80% | 67.46% | 99.66% | 99.83% | 100.00% |  |
| C-TAN/BJ202403 | 72.24% | 99.43% | 72.42% | 67.69% | 67.35% | 99.66% | 99.94% | 99.77% |  |
| C-TAN/BJ202404 | 72.18% | 99.49% | 72.48% | 67.75% | 67.41% | 99.72% | 99.89% | 99.94% |  |
| C-TAN/BJ202405 | 72.24% | 99.43% | 72.54% | 67.80% | 67.46% | 99.66% | 99.83% | 99.89% |  |
| C-TAN/BJ202401 | 99.79% | 84.71% | 82.68% | 83.71% | 87.27% | 84.80% | 99.73% | 85.35% | H |
| C-TAN/BJ202402 | 85.35% | 97.28% | 81.60% | 88.33% | 87.47% | 97.54% | 85.28% | 99.97% |  |
| C-TAN/BJ202403 | 85.59% | 97.52% | 81.98% | 88.40% | 87.75% | 97.54% | 85.52% | 99.14% |  |
| C-TAN/BJ202404 | 85.35% | 97.32% | 81.63% | 88.36% | 87.47% | 97.57% | 85.28% | 100.00% |  |
| C-TAN/BJ202405 | 85.32% | 97.14% | 81.53% | 88.26% | 87.37% | 97.40% | 85.25% | 99.83% |  |
| C-TAN/BJ202401 | 99.77% | 96.29% | 37.56% | 99.54% | 99.77% | 97.62% | 99.77% | 99.77% | P |
| C-TAN/BJ202402 | 99.77% | 96.41% | 37.56% | 99.54% | 99.77% | 97.62% | 99.77% | 99.88% |  |
| C-TAN/BJ202403 | 99.71% | 96.23% | 37.61% | 99.48% | 99.71% | 97.62% | 99.71% | 99.71% |  |
| C-TAN/BJ202404 | 99.94% | 96.46% | 37.61% | 99.71% | 99.94% | 97.80% | 99.94% | 99.94% |  |
| C-TAN/BJ202405 | 99.88% | 96.41% | 37.56% | 99.65% | 99.88% | 97.74% | 99.88% | 99.88% |  |

Note: The similarity matrix was computed after sequence alignment.

**Table S6 Analysis of HAdV recombination events with seven algorithms**

|  |  |  | **Methods** | | | | | |  | **Breakpoint (bp)** | |
| --- | --- | --- | --- | --- | --- | --- | --- | --- | --- | --- | --- |
| **Recombination sequence** | **Major** | **Minor** | **RDP** | **GENECONV** | **BootScan** | **MaxChi** | **Chimaera** | **SiScan** | **3Seq** | **Start** | **End** |
| C-TAN/BJ202401 | C1 | C5 | + | + | + | + | + | + | + | 8134 | 10418 |
| C-TAN/BJ202402 | C2 | C1 | + | + | + | + | - | + | + | 11320 | 14921 |
| C-TAN/BJ202403 | C2 | C1 | + | + | + | + | + | + | + | 64 | 2392 |
| C-TAN/BJ202404 | C2 | C5 | + | + | + | + | + | + | + | 1 | 10112 |

Notes:

+: Support this recombination event

-: Deny this recombination event

Recombination events were identified using the seven algorithms implemented in RDP4.

**Table S7 Statistical analysis of synonymous and nonsynonymous mutation frequencies in Beijing HAdV-C strains.**

|  | **Student**'**s t-test** | **t-statistic** | **Summary** | ***P*-value** |
| --- | --- | --- | --- | --- |
| **N/S ratio** | Immune regulatory vs. Capsid | 12.9273 | *** | <0.001 |
|  | Immune regulatory vs. Replication Regulatory | 12.5892 | *** | <0.001 |
|  | Immune regulatory vs. Metabolic Regulatory | -0.1636 | ns | 0.8741 |
|  | Immune regulatory vs.  Virion Assembly | 21.9547 | *** | <0.001 |
|  | Immune regulatory vs.  DNA Packaging | 23.7382 | *** | <0.001 |
|  | Capsid vs.  Replication Regulatory | 4.4134 | ** | 0.0022 |
|  | Capsid vs.  Metabolic Regulatory | -0.6219 | ns | 0.5513 |
|  | Capsid vs.  Virion Assembly | 13.5305 | *** | <0.001 |
|  | Capsid vs.  DNA Packaging | 16.8069 | *** | <0.001 |
|  | Replication Regulatory vs. Metabolic Regulatory | -0.8249 | ns | 0.4333 |
|  | Replication Regulatory vs. Virion Assembly | 4.3818 | ** | 0.0023 |
|  | Replication Regulatory vs. DNA Packaging | 8.8544 | *** | <0.001 |
|  | Metabolic Regulatory vs. Virion Assembly | 1.0436 | ns | 0.3272 |
|  | Metabolic Regulatory vs. DNA Packaging | 1.3339 | ns | 0.2190 |
|  | Virion Assembly vs. DNA Packaging | 6.2757 | *** | <0.001 |

Notes:

Student's t-test. Significance thresholds: **P* ≤ 0.05, ***P* ≤ 0.01, ****P* ≤ 0.001, ns = not significant.

**Table S8 Mutation statistics of E1A-26K and E1B-55K proteins in sequences used for selection analysis**

| **Accession number** | **Type** | **E1A Count** | **E1A Mutations** | **E1B-55K Count** | **E1B Mutations** |
| --- | --- | --- | --- | --- | --- |
| C-TAN/BJ202401 | C1 | 6 | D68E, L81F, A158T, M163L, R218Q, P230S | 3 | A53-, F114S, V120A |
| MH183293.1 | C1 | 3 | A29T, A158T, R218Q | 3 | A53-, F114S, V120A |
| MN513341.1 | C1 | 3 | A29T, A158T, R218Q | 3 | A53G, F114S, V120A |
| MN513344.1 | C1 | 3 | A29T, A158T, R218Q | 6 | F88Y, N98T, F114C, V120A, T124A, V136I |
| MW686834.1 | C1 | 0 |  | 2 | F114S, V120A |
| MW686838.1 | C1 | 0 |  | 1 | F114S |
| NMDCN0007NAF | C1 | 3 | A158T, T172E, R218Q | 6 | F88Y, N98T, F114C, V120A, T124A, V136I |
| NMDCN0007NAG | C1 | 2 | A158T, R218Q | 1 | F114S |
| NMDCN0007NAI | C1 | 2 | A158T, R218Q | 1 | F114S |
| NMDCN0007NAJ | C1 | 3 | A29T, A158T, R218Q | 7 | A53-, F88Y, N98T, F114C, V120A, T124A, V136I |
| OM112290.1 | C1 | 0 |  | 2 | F114S, V120A |
| OM112294.1 | C1 | 3 | A29T, A158T, R218Q | 1 | F114S |
| OQ518300.1 | C1 | 0 |  | 1 | F114S |
| OQ518310.1 | C1 | 0 |  | 2 | F114S, V120A |
| OQ518311.1 | C1 | 6 | D68E, L81F, A158T, M163L, R218Q, P230S | 1 | F114S |
| OQ518338.1 | C1 | 1 | L81F | 1 | F114S |
| OQ518347.1 | C1 | 0 |  | 2 | F114S, V120A |
| OR735184.1 | C1 | 5 | D68E, L81F, A158T, R218Q, P230S | 2 | A53-, F114S |
| OR735199.1 | C1 | 6 | D68E, L81F, A158T, M163L, R218Q, P230S | 3 | A53-, F114S, V120A |
| OR735206.1 | C1 | 3 | A29T, A158T, R218Q | 1 | F114S |
| OR753096.1 | C1 | 3 | A158T, T172E, R218Q | 3 | A53-, F114S, V120I |
| OR753107.1 | C1 | 6 | D68E, L81F, A158T, M163L, R218Q, P230S | 2 | A53-, F114S |
| OR753113.1 | C1 | 6 | D68E, L81F, A158T, M163-, R218Q, P230S | 3 | A53-, F114S, V120A |
| OR753115.1 | C1 | 3 | A29T, A158T, R218Q | 1 | F114S |
| OR753130.1 | C1 | 3 | A29T, A158T, R218Q | 1 | F114S |
| OR753138.1 | C1 | 6 | D68E, L81F, A158T, M163L, R218Q, P230S | 3 | A53-, F114S, V120A |
| OR777163.1 | C1 | 6 | D68E, L81F, A158T, M163L, R218Q, P230S | 3 | A53-, F114S, V120A |
| OR777176.1 | C1 | 3 | A29T, A158T, R218Q | 7 | A53-, F88Y, N98T, F114C, V120A, T124A, V136I |
| OR777177.1 | C1 | 2 | A158T, R218Q | 3 | A53-, F114S, V120A |
| OR777182.1 | C1 | 0 |  | 2 | F114S, V120A |
| OR777192.1 | C1 | 0 |  | 1 | F114S |
| AC_000007.1 | C2 | 6 | D68E, L81F, A158T, M163L, R218Q, P230S | 3 | A53-, F114S, V120A |
| KX384959.1 | C2 | 6 | A29V, D68E, L81F, A158T, R218Q, P230S | 3 | A53-, F114S, V120A |
| MF044052.1 | C2 | 3 | D68E, A158T, R218Q | 6 | F88Y, N98T, F114C, V120A, T124A, V136I |
| MN513342.1 | C2 | 3 | A29T, A158T, R218Q | 3 | A53-, F114S, V120A |
| OQ518261.1 | C2 | 3 | A29T, A158T, R218Q | 2 | F114S, V120A |
| OQ518272.1 | C2 | 3 | A29T, A158T, R218Q | 1 | F114S |
| OR735186.1 | C2 | 5 | D68E, L81F, A158T, R218Q, P230S | 3 | A53-, F114S, V120A |
| OR735196.1 | C2 | 3 | A29T, A158T, R218Q | 7 | A53-, F88Y, N98T, F114C, V120A, T124A, V136I |
| OR753114.1 | C2 | 3 | A158T, T172E, R218Q | 2 | F114S, V120A |
| OR777220.1 | C2 | 6 | D68E, L81F, A158T, M163L, R218Q, P230S | 3 | A53-, F114S, V120A |
| AC_000008.1 | C5 | 2 | A158T, R218Q | 7 | A53-, F88Y, N98T, F114C, V120A, T124A, V136I |
| KX868466.2 | C5 | 5 | D68E, L81F, A158T, R218Q, P230S | 3 | A53-, F114S, V120A |
| MW686848.1 | C5 | 5 | D68E, L81F, A158T, R218Q, P230S | 7 | A53-, F88Y, N98T, F114C, V120A, T124A, V136I |
| NMDCN0003GMS | C5 | 3 | A29T, A158T, R218Q | 2 | F114S, V120A |
| NMDCN0007NAK | C5 | 3 | A158T, T172E, R218Q | 6 | F88Y, N98T, F114C, V120A, T124A, V136I |
| OQ518274.1 | C5 | 3 | A29T, A158T, R218Q | 7 | A53-, F88Y, N98T, F114C, V120A, T124A, V136I |
| OQ518277.1 | C5 | 3 | A29T, A158T, R218Q | 7 | A53-, F88Y, N98T, F114C, V120A, T124A, V136I |
| OR728260.1 | C5 | 6 | D68E, L81F, A158T, M163-, R218Q, P230S | 3 | A53-, F114S, V120A |
| OR735179.1 | C5 | 2 | A29T, A158T | 1 | F114S |
| OR753099.1 | C5 | 5 | D68E, L81F, A158T, R218Q, P230S | 3 | A53-, F114S, V120A |
| OR753108.1 | C5 | 5 | D68E, L81F, A158T, R218Q, P230S | 3 | A53-, F114S, V120A |
| OR777154.1 | C5 | 6 | D68E, L81F, A158T, M163L, R218Q, P230S | 3 | A53-, F114S, V120A |
| OR777173.1 | C5 | 6 | A29T, D68E, L81F, A158T, R218Q, P230S | 3 | A53-, F114S, V120A |
| FJ349096.1 | C6 | 6 | D68E, L81F, A158T, M163L, R218Q, P230S | 3 | A53-, F114S, V120A |
| LC068715.1 | C6 | 6 | D68E, L81F, A158T, M163L, R218Q, P230S | 3 | A53-, F114S, V120A |
| OR753103.1 | C6 | 4 | D68E, L81F, A158T, R218Q | 6 | F88Y, N98T, F114S, V120A, T124A, V136I |
| LC062717.1 | C57 | 3 | A29T, A158T, R218Q | 7 | A53-, F88Y, N98T, F114C, V120A, T124A, V136I |
| LC068707.1 | C57 | 3 | A29T, A158T, R218Q | 7 | A53-, F88Y, N98T, F114C, V120A, T124A, V136I |
| C-TAN/BJ202402 | C108 | 6 | D68E, L81F, A158T, M163L, R218Q, P230S | 3 | A53-, F114S, V120A |
| C-TAN/BJ202403 | C108 | 0 |  | 2 | A53-, F114S |
| C-TAN/BJ202404 | C108 | 2 | A158T, R218Q | 7 | A53-, F88Y, N98T, F114C, V120A, T124A, V136I |
| C-TAN/BJ202405 | C108 | 3 | A29T, A158T, R218Q | 2 | F114S, V120A |
| C_AA104072.1 | C108 | 3 | A29T, A158T, R218Q | 7 | A53-, F88Y, N98T, F114C, V120A, T124A, V136I |
| C_AA104083.1 | C108 | 3 | A158I, T172E, R218Q | 2 | A53-, F114S |
| MW686840.1 | C108 | 0 |  | 2 | A53-, F114S |

Notes:

In this table, AF534906.1 was used as the reference sequence.

-: There is a gap.

**Table S9 Positively selected sites in 23 genes across 66 HAdV-C strains.**

| **Protein ID** | **Length (aa)** | **Function** | **Positively selected sites** | **Substitution ratio** |
| --- | --- | --- | --- | --- |
| E1a-6KD AAQ10564 | 55 | Replication regulatory | 41 N 1.000**,47 G 1.000**,50 K 1.000** | 0.036 |
| E4-20KD AAQ10573 | 182 | Metabolic regulatory Replication regulatory Immune regulatory | 157 I 1.000**,158 A 1.000**,165 R 1.000**,177 C 1.000**,180 V 1.000**,181 T 1.000** | 0.033 |
| E2B-14KD AAQ10567 | 132 | Unknow | 16 R 0.994**,58 K 1.000**,100 T 1.000**,102 L 1.000** | 0.030 |
| E1a-26KD AAQ10563 | 243 | Replication regulatory Immune regulatory | 29 A 1.000**,68 D 1.000**,81 L 1.000**,158 A 1.000**,163 M 1.000**,172 T 1.000**,218 R 1.000**,230 P 1.000** | 0.029 |
| E1a-32KD AAQ10538 | 289 | Replication regulatory Immune regulatory | 29 A 1.000**,68 D 1.000**,81 L 1.000**,149 Q 1.000**,204 A 1.000**,218 T 1.000**,264 R 1.000**,276 P 1.000** | 0.028 |
| E4-32KD AAQ10562 | 283 | Metabolic regulatory Replication regulatory Immune regulatory | 122 E 1.000**,258 I 1.000**,259 A 1.000**,266 R 1.000**,278 C 1.000**,281 V 1.000**,282 T 1.000** | 0.025 |
| E4-27KD AAQ10571 | 245 | Metabolic regulatory Replication regulatory Immune regulatory | 220 I 1.000**,221 A 1.000**,228 R 1.000**,240 C 1.000**,243 V 1.000**,244 T 1.000** | 0.024 |
| E1B 1.31kb mRNA AAQ10540 | 97 | Unknow | 53 A 1.000**,84 E 1.000** | 0.021 |
| Ⅸ AAQ10541 | 140 | Immune regulatory Viron assembly | 3 T 1.000**,123 E 1.000** | 0.014 |
| E1B-55KD AAQ10566 | 500 | Replication regulatory Immune regulatory | 53A 1.000**,88 F 1.000**,98 N 1.000**,114F 1.000**,120 V 1.000**,124 T 1.000**,136 V 1.000** | 0.014 |
| DNA Pol AAQ10543 | 1198 | Replication regulatory | 33 P 0.997**,56 R 1.000**,64 P 0.999**,176 V 0.994**,321 V 1.000**,322 D 1.000**,358 M 1.000**,369 V 0.990**,376 A 0.999**,405 T 1.000**,738 I 1.000**,793 H 1.000**,897 Q 1.000**,901 S 0.997**,926 T 1.000** | 0.013 |
| E3-12kD AAQ10568 | 107 | Immune regulatory | 8 Q 0.997** | 0.009 |
| E2a-25KD AAQ10557 | 227 | Viron assembly | 49 A 0.993**,60 S 0.992** | 0.009 |
| E4-28.6KD AAQ10570 | 255 | Metabolic regulatory | 70 N 0.991**,122 E 1.000** | 0.008 |
| L1-16.7KD AAQ10545 | 149 | DNA Packaging | 14 V 1.000** | 0.007 |
| E1B 1.26kb mRNA AAQ10565 | 160 | Immune regulatory | 53 A 1.000** | 0.006 |
| Penton base AAQ10548 | 574 | Capsid | 157 K 1.000**,420 A 0.997**,458 S 0.998** | 0.005 |
| L1-47KD AAQ10546 | 415 | DNA Packaging  Viron assembly | 30 S 1.000**,34 S 0.992** | 0.005 |
| pVI AAQ10552 | 250 | Viron assembly  Replication regulatory | 124 L 0.999** | 0.004 |
| L5-90KD AAQ10556 | 807 | Viron assembly | 240 C 0.994**,290 Q 1.000**,354 K 0.994** | 0.004 |
| Ⅲa AAQ10547 | 585 | DNA Packaging  Viron assembly | 463 A 1.000**,474 L 0.999** | 0.003 |
| pTP AAQ10544.2 | 670 | Replication regulatory | 200 R 0.991**,390 V 0.999** | 0.003 |
| IVa2 AAQ10542.2 | 449 | Replication regulatory  DNA Packaging | 137 R 0.993** | 0.002 |


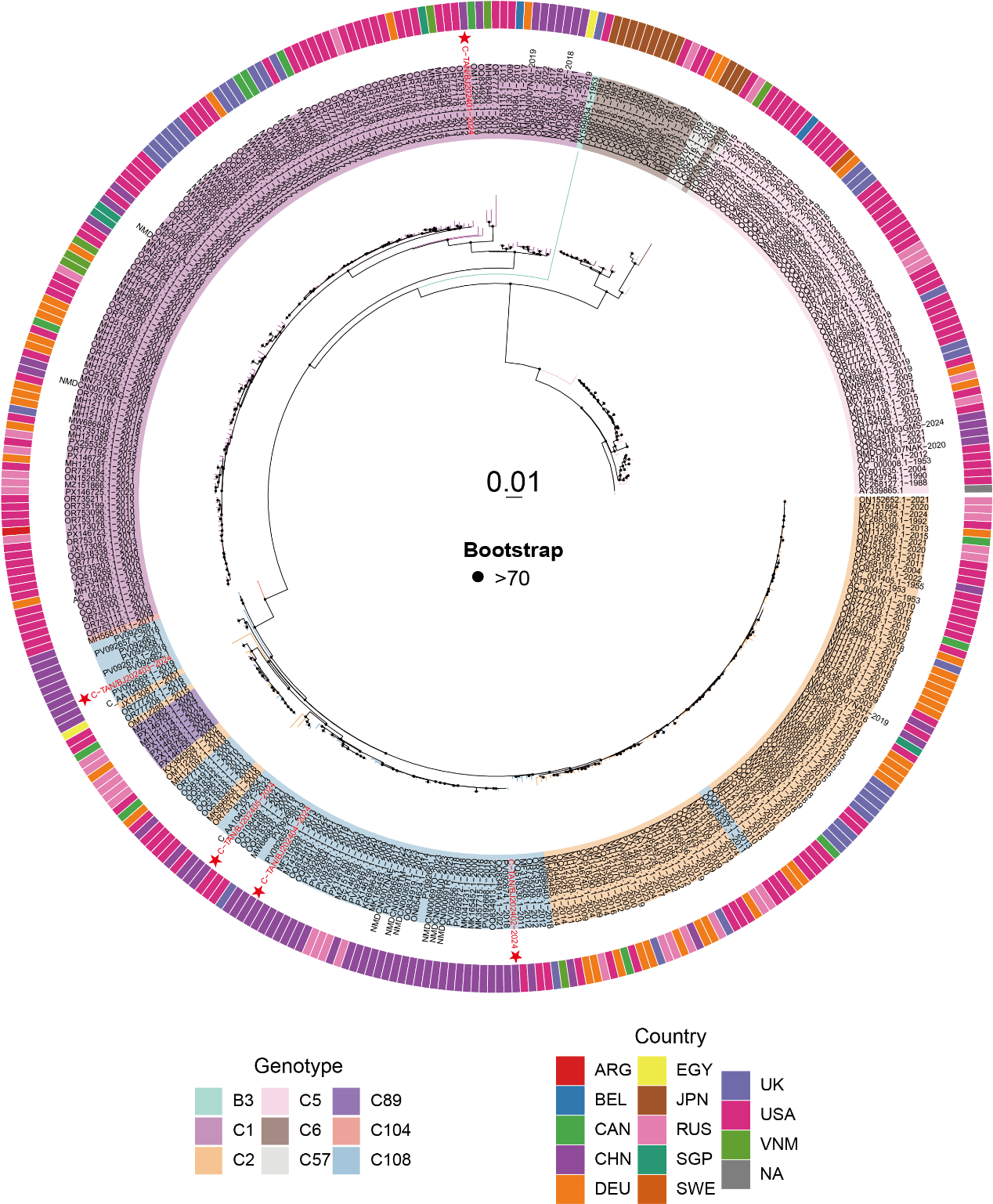


**Figure S1 Phylogenetic tree based on the whole-genome sequence**

The ML Phylogenetic tree of whole-genome sequence, including eight genotype of HAdV-C1, HAdV-C2, HAdV-C5, HAdV-C6, HAdV-C57, HAdV-C89, HAdV-C104, and HAdV-C108. The five HAdV-C strains were highlighted in red color and stars. Colors in the outer ring represent countries, while the inner sectors are shown in different colors to represent genotypes.


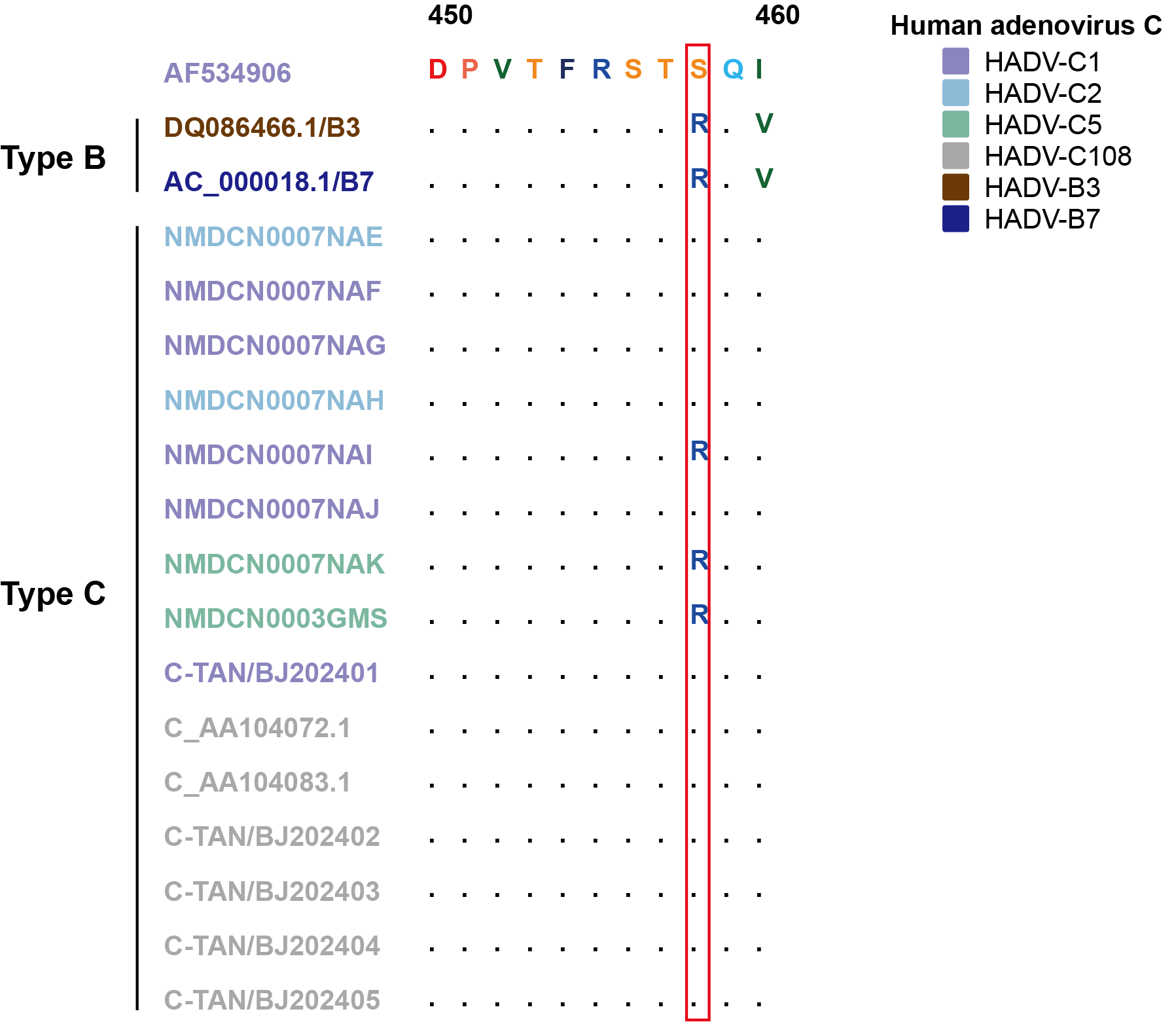


**Figure S2 Protein sequence alignment of the Penton base**.

Protein sequence alignment was performed on a subset of sequences originating from China in this study. Sites where substitutions occurred are highlighted with the red rectangles.

**
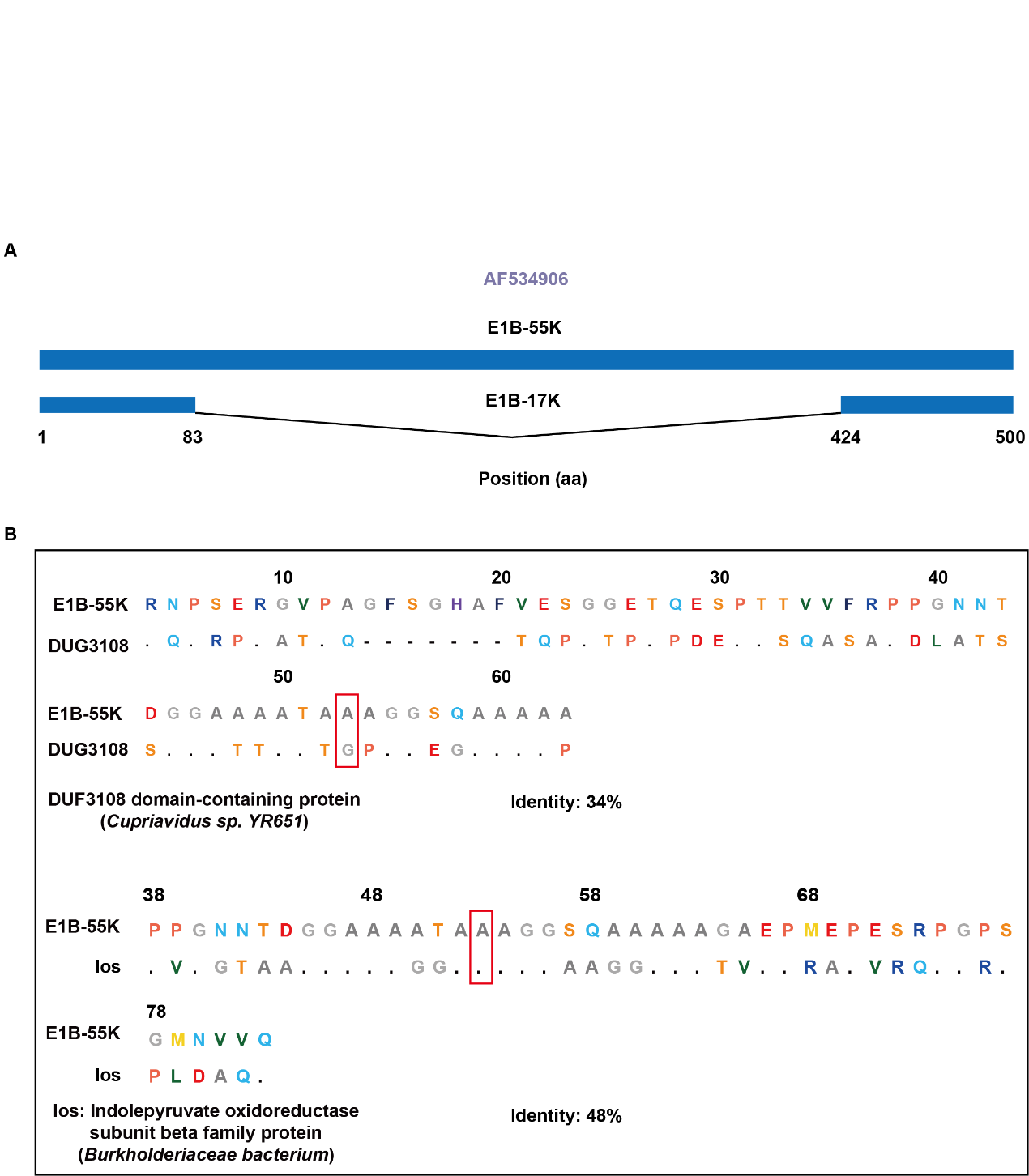
**

**Figure S3 Homology alignment of the E1B-55K protein**

(**A**) Alternative splicing of clade C1 E1B-55K gives rise to E1B-160R. (**B**) Sequence alignment of E1B-55K with homologous proteins from two pathogenic bacteria. Identical amino acids are denoted by dots. The red rectangles highlight the residue corresponding to the positive selection site A53 in E1B-55K.

**
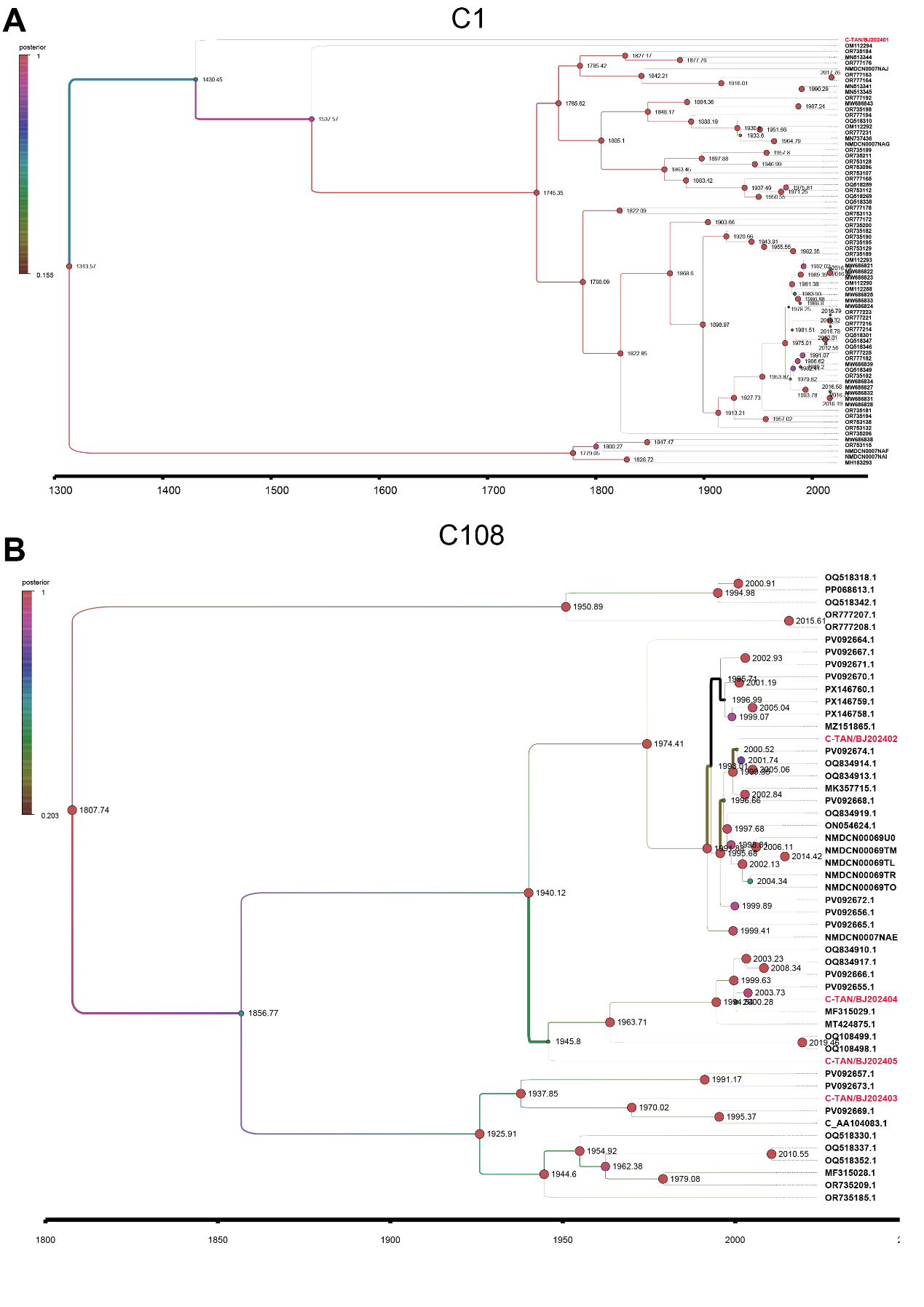
Figure S4 Bayesian estimation of the time to the most recent common ancestor for types C1 and C108**

The red dots represent the major evolutionary divergences, and the 95% highest posterior density (HPD) range for the time to tMRCA is presented. The sequences described in this study is indicated in red. **(A)** Bayesian estimation of the time to the most recent common ancestor for type C1. **(B)** Bayesian estimation of the time to the most recent common ancestor for type C108.

**References**

Baele G, Lemey P, Bedford T *et al.* Improving the accuracy of demographic and molecular clock model comparison while accommodating phylogenetic uncertainty. *Mol Biol Evol* 2012;**29**:2157–67. <https://doi.org/10.1093/molbev/mss084>

Kass RE, Raftery AE. Bayes Factors. *Journal of the American Statistical Association* 1995;**90**:773–95. <https://doi.org/10.1080/01621459.1995.10476572>
